# Factors Associated with Malaria Parasitemia, Anemia and Serological Responses in a Spectrum of Epidemiological Settings in Uganda

**DOI:** 10.1371/journal.pone.0118901

**Published:** 2015-03-13

**Authors:** Adoke Yeka, Joaniter Nankabirwa, Arthur Mpimbaza, Ruth Kigozi, Emmanuel Arinaitwe, Chris Drakeley, Bryan Greenhouse, Moses R. Kamya, Grant Dorsey, Sarah G. Staedke

**Affiliations:** 1 Makerere University School of Public Health, College of Health Sciences, Kampala, Uganda; 2 Department of Medicine, Makerere University College of Health Sciences, Kampala, Uganda; 3 Child Health and Development Centre, Makerere University College of Health Sciences, Kampala, Uganda; 4 Infectious Diseases Research Collaboration, Kampala, Uganda; 5 London School of Hygiene and Tropical Medicine, London, United Kingdom; 6 Department of Medicine, University of California San Francisco, San Francisco, United States of America; Singapore Immunology Network, Agency for Science, Technology and Research (A*STAR), SINGAPORE

## Abstract

**Background:**

Understanding the current epidemiology of malaria and the relationship between intervention coverage, transmission intensity, and burden of disease is important to guide control activities. We aimed to determine the prevalence of anemia, parasitemia, and serological responses to *P*. *falciparum* antigens, and factors associated with these indicators, in three different epidemiological settings in Uganda.

**Methods and Findings:**

In 2012, cross-sectional surveys were conducted in 200 randomly selected households from each of three sites: Walukuba, Jinja district (peri-urban); Kihihi, Kanungu district (rural); and Nagongera, Tororo district (rural) with corresponding estimates of annual entomologic inoculation rates (aEIR) of 3.8, 26.6, and 125.0, respectively. Of 2737 participants, laboratory testing was done in 2227 (81.4%), including measurement of hemoglobin, parasitemia using microscopy, and serological responses to *P*. *falciparum* apical membrane antigen 1 (AMA-1) and merozoite surface protein 1, 19 kilodalton fragment (MSP-1_19_). Analysis of laboratory results was restricted to 1949 (87.5%) participants aged ≤ 40 years. Prevalence of anemia (hemoglobin < 11.0 g/dL) was significantly higher in Walukuba (18.9%) and Nagongera (17.4%) than in Kihihi (13.1%), and was strongly associated with decreasing age for those ≤ 5 years at all sites. Parasite prevalence was significantly higher in Nagongera (48.3%) than in Walukuba (12.2%) and Kihihi (12.8%), and significantly increased with age to 11 years, and then significantly decreased at all sites. Seropositivity to AMA-1 was 53.3% in Walukuba, 63.0% in Kihihi, and 83.7% in Nagongera and was associated with increasing age at all sites. AMA-1 seroconversion rates strongly correlated with transmission intensity, while serological responses to MSP-1_19_ did not.

**Conclusion:**

Anemia was predominant in young children and parasitemia peaked by 11 years across 3 sites with varied transmission intensity. Serological responses to AMA-1 appeared to best reflect transmission intensity, and may be a more accurate indicator for malaria surveillance than anemia or parasitemia.

## Introduction

Malaria remains an important public health problem in sub-Saharan Africa, and is responsible for over 10% of the overall disease burden [1]. In the past decade, increased donor financing and widespread scale-up of malaria control measures, including distribution of long-lasting insecticide-treated bed nets (LLINs), indoor residual spraying (IRS) with insecticides, intermittent presumptive treatment in pregnancy (IPTp), and prompt and effective treatment with highly efficacious artemisinin-based combination therapies (ACTs), have substantially reduced the malaria burden in several countries [2–7]. However, these gains have not been consistent across Africa [8], and malaria-associated morbidity and mortality remains high in some countries, including Uganda [9–11].

In 2012, Uganda was ranked fourth highest in number of malaria cases reported globally, and sixth in number of malaria-associated deaths [12]. Although the epidemiology of malaria varies widely, some of the highest levels of malaria transmission in the world have been recorded in Uganda, and much of the population lives in high transmission areas [11]. Uganda has made mixed progress towards implementing malaria control interventions in the past decade. Ownership of at least one insecticide treated net (ITN) per household increased substantially from 16% in 2006 to 60% in 2011, but national IRS coverage remains low (6–7%). In 2011, the proportion of pregnant women receiving two or more doses of intermittent preventive treatment (IPTp) was only 25%, while the proportion of febrile children treated with artemether-lumefantrine (AL), the first-line recommended treatment for uncomplicated malaria, was 44%, up from 23% in 2006 [13,14]. Data documenting the impact of scaling-up of control interventions on indicators of malaria burden in Uganda are limited. Only IRS has been associated with a reduction in malaria morbidity [15], and this benefit has been transient in areas where IRS has not been sustained [16]. Some evidence suggests that despite modest improvements in coverage of malaria control interventions, the burden of malaria may be increasing in certain areas of Uganda [17].

Monitoring and evaluation of malaria control activities relies heavily on large, nationally representative cross-sectional surveys. In Uganda, these include Demographic Health Surveys (DHS) which have been conducted every 5 years since 2001 and a single Malaria Indicator Survey (MIS) conducted in 2009. Although all these surveys include estimates of coverage of malaria control interventions, indicators of malaria burden are limited to anemia testing and, only in the 2009 MIS, estimates of parasitemia. The annual entomological inoculation rate (aEIR) is generally considered the ‘gold-standard’ measure of malaria endemicity and transmission intensity, but is highly labour-intensive and rarely measured [18]. In areas of stable malaria transmission, anemia has been used to monitor the impact of control interventions [19–21]. Parasite prevalence in children aged 2–10 years provides an indirect measure of transmission intensity across a range of malaria endemicities and is the most frequently measured malariometric index [22,23]. More recently, serological responses to malaria antigens have gained momentum as indicators of malaria transmission and infection risk, as seroprevalence reflects cumulative malaria exposure [24]. Recent studies have capitalized on the relationship between *P*. *falciparum* exposure and antibody responses to show that it is possible to estimate transmission intensity in a community by calculating seroconversion rates using age specific prevalence of specific antimalarial antibodies, including those to apical membrane antigen 1 (AMA-1) and merozoite surface protein 1, 19 kilodalton fragment (MSP-1_19_) [25–27].

To better understand the current epidemiology of malaria in Uganda, cross-sectional surveys to estimate coverage levels of control interventions and key malaria indicators, including anemia, parasitemia, and serological responses to AMA-1 and MSP-1_19_ were conducted in three sub-counties with varying levels of transmission intensity based on concurrently measured estimates of aEIR.

## Methods

### Study design

This was a cross-sectional community survey conducted in three different epidemiological settings in Uganda. A total of 200 households were recruited in each site, using a list of households randomly generated from a census database. The surveys consisted of a household questionnaire and biomarker survey. Finger-prick blood samples, obtained from all children under fifteen years of age and from a random selection of age stratified adults, were collected for measurement of hemoglobin, thick and thin blood smear, rapid diagnostic test (RDT) for malaria, and to save on filter paper. The protocol for this trial is available as supporting information (S1 Protocol).

### Ethics statement

Ethical approval was obtained from the Makerere University School of Medicine Research and Ethics Committee (REC REF 2011–203), the Uganda National Council for Science and Technology (HS 1074), the London School of Hygiene & Tropical Medicine Ethics Committee (Reference 6012), and the University of California, San Francisco Committee on Human Research (Reference 027911). For all households fulfilling the selection criteria, written consent to participate in the study was sought. Written informed consent was obtained from the next of kin, caretakers, or guardians on behalf of the children enrolled in the study. Furthermore, written assent was obtained from children aged 8 years and above to participate in the study.

### Study sites

**Fig 1:**
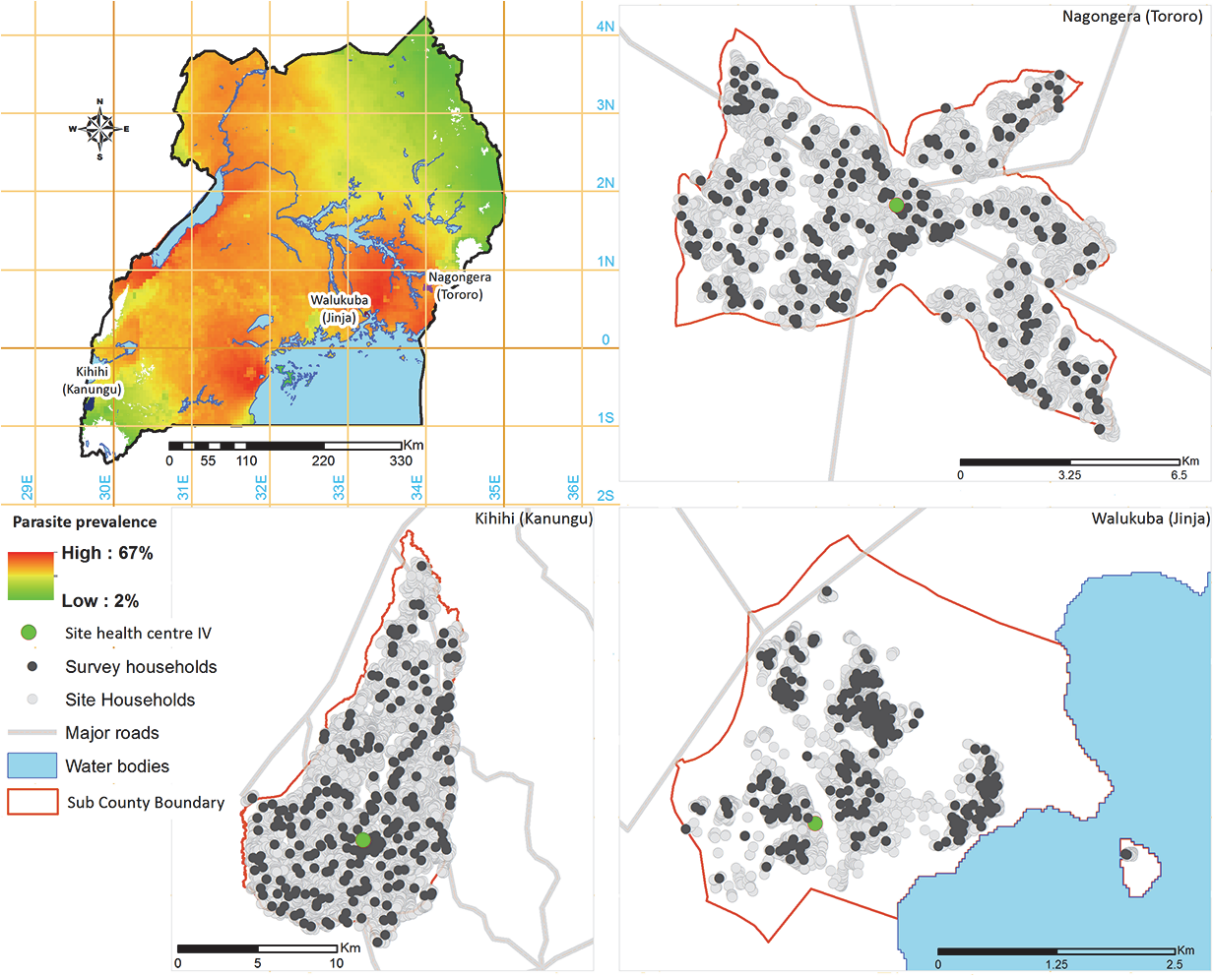
Map of study sites in Uganda.

Cross-sectional surveys were conducted between January and June 2012 in three sub-counties purposively chosen to represent varied malaria transmission settings in Uganda: Walukuba, Jinja district; Kihihi, Kanungu district; and Nagongera, Tororo district (Fig. 1). In each sub-county, entomological surveys were conducted from October 2011 – September 2012 using monthly human landing catches to generate estimates of aEIR as previously described [28]. Malaria transmission at all three sites was perennial, with 2 annual peaks following the rainy seasons. Walukuba is a peri-urban area near Lake Victoria in the south-central part of the country with an estimated aEIR of 3.8 infectious bites per person per year. Kihihi is a rural area in the south-western part of the country, with an estimated aEIR of 26.6. Nagongera is a rural area in the south-eastern part of the country near the Kenyan border, with an estimated aEIR of 125.0. Key malaria control interventions in all three districts include use of LLINs, malaria case management with ACTs, and IPTp with sulfadoxine-pyrimethamine. A single round of IRS using lambda-cyhalothrin was conducted in Kanungu district in 2007 and a mass, community-based campaign to distribute free ITNs was conducted in Tororo district in January 2011.

### Enumeration and mapping of households

In 2011, all households and other key features in the study areas were enumerated and mapped to generate a sampling frame for the surveys. A household was defined as any single permanent or semi-permanent dwelling structure acting as the primary residence for a person or group of people that generally cook and eat together. Household locations were mapped using hand-held eTrex global positioning system (GPS) receivers (Garmin Ltd., Olathe, KS) and readings were taken from the door of the household, if possible, or from a point that was most representative of the household.

### Recruitment and enrolment

We enrolled 200 households from each site using a list randomly generated from the household enumeration database using computer-generated random numbers. Study personnel conducted door-to-door recruitment to identify those households with at least one adult household resident in sequential order according to the recruitment list. Residents not home during the initial contact were re-visited on at least three other occasions before eliminating them from the sample selection process.

When a household was identified, study personnel briefly described the purpose of the study to the head of the household or any adult household member present at home in the appropriate language. Households were included if: 1) an adult aged 18 years or older was the head of the household, and 2) the head of the household agreed to provide informed consent. Households were excluded if: 1) no adult resident was at home on more than 3 visits, and 2) the household was vacant. For all households fulfilling the selection criteria, written consent to participate in the study was sought.

### Study procedures

Upon enrolment, a household questionnaire, based on the Malaria Indicator Survey questionnaire developed by the Roll Back Malaria Monitoring and Evaluation Reference Group [29], was administered to heads of households or their designate. The questionnaire was used to obtain information on all household members, including age, gender, and relationship to the head of the household. It was also used to collect information on ownership and use of bed nets, and proxy indicators of wealth including ownership of assets and land, household construction, and food security. Finger-prick blood samples were obtained from all children under fifteen years of age, and from one randomly selected household member within the following age categories: 15–24 years, 25–34 years, 35–44 years, 45–54 years, and > 55 years. Blood samples were used for measurement of hemoglobin, thick and thin blood smear, and storage on filter paper.

### Laboratory evaluations

Thick and thin blood smears were stained with 2% Giemsa for 30 minutes. Thick smears were evaluated for the presence of parasitemia (asexual forms only) and gametocytes. Parasite densities were calculated by counting the number of asexual parasites per 200 leukocytes (or per 500, if the count was less than 10 parasites or gametocytes per 200 leukocytes), assuming a leukocyte count of 8,000/*u*l. A smear was considered negative after reviewing 100 high-powered fields. All positive thick blood smears had corresponding thin smears reviewed for species identification. For quality control, all slides were read by a second microscopist and a third reviewer was used to settle any discrepant readings. Hemoglobin concentration was assessed using a battery-operated portable hemoglobinometer (HemoCue Ltd., Angelholm, Sweden) and estimated to an accuracy of 1 g/dL.

Blood spots were collected onto filter papers (Whatman 3MM; Whatman, Maidstone, UK), allowed to dry overnight and stored at -20°C with dessicant for serology studies. The recombinant proteins *P*. *falciparum* AMA-1 (3D7) and MSP-1_19_ (Wellcome genotype) were used as antigens in indirect ELISA as previously described [30]. Briefly, antigens were coated on plates at the concentration of 0.5 mg/mL in coating buffer and incubated at 4^o^C overnight. The plates were washed in PBST, and blocked with 1% (w/v) skimmed milk solution for three hours. After washing, samples were added in duplicate at a final dilution of 1:1,000 to each plate together with a pool of hyper immune serum and the plates were incubated overnight at 4^o^C. The plates were washed and 50 μl of HRP-conjugated rabbit anti human IgG (DAKO, #P0214) were added into each well and incubated for three hours. After a further series of washes, substrate solution (OPD, Sigma #P8287, in PBS) was added and the reaction was allowed to develop for 15–20 min before addition of stopping solution (2M H2SO4). The optical density was read using an ELISA reader at 450 nm.

### Data management and statistical analysis

Data were collected using hand-held computers which were programmed to include range checks, structure checks and internal consistency checks. All statistical analyses were carried out using Stata version 12 (STATA Corporation, College Station, TX). Malaria indicators of interest were; 1) anemia defined as hemoglobin less than 11 g/dl, 2) parasitemia defined on the basis of expert microscopy results as presence of any asexual parasites on thick blood smear, 3) AMA-1 antibody seropositivity and, 4) MSP-1_19_ antibody seropositivity. Raw optical density (OD) measurements were averaged and normalized against the positive control samples on each plate. The cut-off for seropositivity was calculated, as previously described with a separate cut-off value generated for each antigen [27]. Seroconversion rates were estimated by fitting a simple reversible catalytic model to seroprevalence data for each antigen [24]. Analyses of malaria indicators were limited to study participants aged 40 years or younger due to the relatively limited number of observations, particularly in one site (Walukuba), and concerns about lack of precision of analyses including participants above 40 years of age. Comparisons between the sites for categorical variables were made using the Chi-squared or Fisher’s exact test and for continuous variables using the t-test. Associations between risk factors of interest and binary malaria indicators were made using multivariate generalized estimating equations stratified by study site with adjustment for clustering of study participants within the same household. In these models the relationships between age and malaria indicators were fitted using linear splines. A household wealth index was created using principal component analysis of data based on household’s dwelling unit ownership of various durable goods, land, and household food security and then categorized into tertiles [31]. Graphical presentation of the relationships between age and both the prevalence of anemia and parasitemia were made using lowess smoothing.

## Results

### Participant characteristics and coverage of control interventions

At total of 200 households were surveyed from each sub-county, including 2737 study participants “Table 1”. The age and gender of participants were similar across the three sites. The majority of households in Nagongera, and over half in Walukuba and Kihihi, owned at least one ITN. However, the proportion of households with at least one ITN per 2 residents was much lower at the 3 sites. Only 5 households in Walukuba reported receiving IRS in the prior 12 months. The proportion of mothers who received ≥ 2 doses of SP for IPTp during their last pregnancy ranged from 35.3% in Walukuba to 52.8% in Kihihi. The use of ACTs for antimalarial treatment was high (≥ 75%) at all sites. A total of 2227 (81.4%) participants underwent laboratory testing, and of these, 1949 (87.5%) aged ≤ 40 years were included in the analysis of laboratory results “Table 2”.

**Table 1:** Characteristics of study sites, households, and participants.

Category	Characteristic	Study Site		
		Walukuba	Kihihi	Nagongera
Transmission intensity	aEIR^a^ using human landing catches (95% CI)	3.8 (0–11.4)	26.6 (7.6–49.4)	125 (72.2–183)
Sample size	Number of household	200	200	200
	Number of study participants	797	948	992
Demographics of survey participants	Female gender, n (%)	399 (50.1%)	499 (52.6%)	537 (54.1%)
	Median age in years (IQR)	18 (6–28)	15 (6–30)	14 (6–32)
Coverage of malaria control interventions	Households with at least 1 ITN, n (%)	115 (57.5%)	102 (51.0%)	157 (78.5%)
	Households with at least 1 ITN per 2 residents, n (%)	57 (28.5%)	34 (17.0%)	71 (35.5%)
	Households reporting IRS in the last 12 months, n (%)	5 (2.5%)	0	0
	IPTp with ≥ 2 doses of SP during last pregnancy^b^, n/N (%)	30/85 (35.3%)	48/91 (52.8%)	31/78 (39.7%)
	Use of ACT if received recent antimalarial treatment^c^, n/N (%)	24/32 (75.0%)	32/39 (82.1%)	49/63 (77.8%)

^a^Annual entomological inoculation rate (measured Oct 2011 – Sept 2012)

^b^Among women who reported giving birth in the previous 4 years

^c^Among children under the age of 5 years with reported fever in the previous 2 weeks

**Table 2:** Estimates of malaria indicators.

Category	Characteristic	Study Site		
		Walukuba	Kihihi	Nagongera
Sample size	Number of participants selected for laboratory testing	631	788	808
	Number of participants selected for laboratory testing ≤ 40 years of age	582	682	685
Anemia testing	Mean hemoglobin g/dL (SD)	12.6 (2.0)	12.8 (1.8)	12.6 (1.9)
	Mean hemoglobin g/dL children < 5 years (SD)	11.3 (1.6)	11.7 (1.5)	10.9 (1.6)
	Hemoglobin < 11 g/dL, n (%)	110 (18.9%)	89 (13.1%)	119 (17.4%)
	Hemoglobin < 11 g/dL children < 5 years, n/N (%)	63/155 (40.7%)	54/183 (29.5%)	78/158 (49.4%)
	Hemoglobin < 8 g/dL, n (%)	7 (1.2%)	4 (0.6%)	3 (0.4%)
Blood smear results	Positive for any asexual parasitemia, n (%)	71 (12.2%)	87 (12.8%)	331 (48.3%)
	Positive for any asexual parasitemia children 2–10 years, n/N (%)	38/233 (16.3%)	50/280 (17.9%)	189/317 (59.6%)
	Geometric mean parasite density/μL	438	881	950
	*P*. *falciparum* infection^a^, n (%)	67 (94.4%)	85 (97.7%)	326 (98.5%)
	Positive for any gametocytes, n (%)	15 (2.6%)	13 (1.9%)	102 (14.9%)
Seroprevalence^b^	AMA-1 seropositive, n/N (%)	309/580 (53.3%)	428/679 (63.0%)	565/675 (83.7%)
	Median AMA-1 antibody titer (IQR)	87 (19–452)	209 (32–913)	709 (182–1887)
	AMA-1 seroconversion rate (95% CI)	0.10 (0.09–0.12)	0.17 (0.14–0.19)	0.42 (0.36–0.49)
	MSP1–^19^ seropositive, n/N (%)	173/544 (31.8%)	344/678 (50.7%)	310/679 (45.7%)
	Median MSP1–^19^ antibody titer (IQR)	66 (40–170)	101 (40–539)	94 (41–458)
	MSP1–^19^ seroconversion rate (95% CI)	0.036 (0.031–0.043)	0.089 (0.078–0.100)	0.064 (0.057–0.073)

^a^Only including those positive for asexual parasites

^b^ No serology results obtain in 16 subjects for AMA-1 and 48 subjects for MSP1–^19^

### Anemia

**Fig 2:**
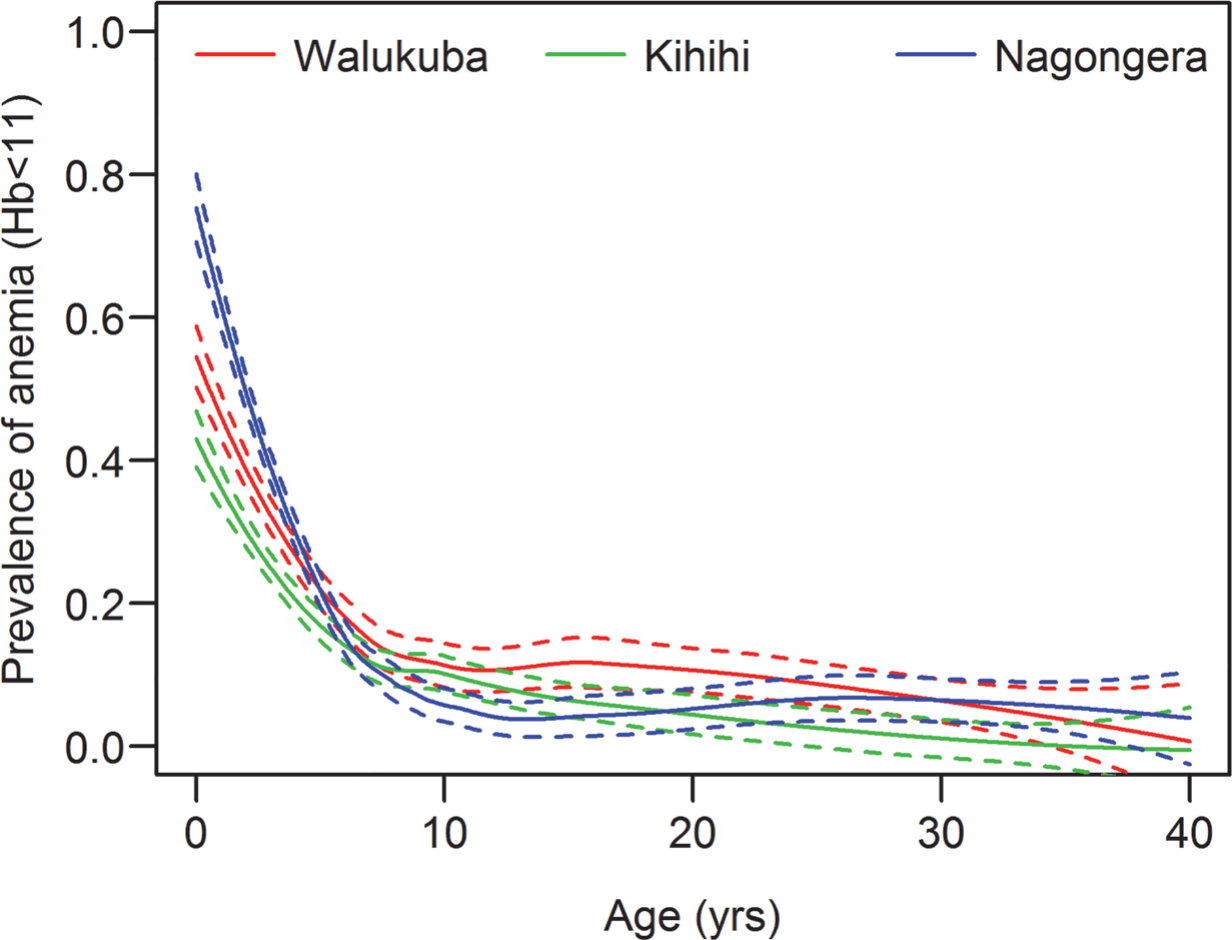
Prevalence of anemia (hemoglobin < 11.0 g/dL) by age, in three different epidemiological settings in Uganda.

Mean hemoglobin levels were generally high (> 12.0 g/dL) and severe anemia (hemoglobin < 8g/dL) was uncommon at all sites “Table 2”. The prevalence of anemia (hemoglobin < 11g/dL) was highest in Walukuba, the site with the lowest EIR “Table 2”. In children under-five, prevalence of anemia was significantly lower in Kihihi (29.5%), than in Walukuba (40.7%, p = 0.03) and Nagongera (49.4%, p<0.001). The probability of anemia was highest in children under-five, decreasing sharply until approximately 10 years of age at all 3 sites (Fig. 2). Considering all sites combined, the prevalence of anemia was 35.7% in those ≤ 5 years of age and 8.0% in those 6–40 years of age (p<0.001). In the adjusted analysis, the prevalence of anemia was most strongly associated with age “Table 3”. For children ≤ 5 years of age, the odds of anemia decreased greatly with increasing age at all the sites (ORs ranging from 0.47 to 0.70 per 1 year increase in age). For participants aged 6–40 years there was a more gradual decrease in the prevalence of anemia in Walukuba (OR 0.96 per 1 year increase) and Kihihi (OR 0.91 per 1 year increase in age). The association between anemia and other factors varied by site; in Kihihi and Nagongera, participants with parasitemia were significantly more likely to be anemic, but ITN use and other factors were not significantly associated. In Walukuba, participants who slept under an ITN the previous night were significantly less likely to be anemic.

**Table 3:** Factors associated with anemia (Hb < 11 g/dL).

Risk factor	Category	Walukuba			Kihihi			Nagongera		
		Hb < 11g/dL	Adjusted OR^a^ (95% CI)	p-value	Hb < 11g/dL	Adjusted OR^a^ (95% CI)	p-value	Hb < 11g/dL	Adjusted OR^a^ (95% CI)	p-value
Age	≤ 5 years	71/183 (38.8%)	0.66 (0.58–0.76) ^b^	<0.001	56/211 (26.5%)	0.70 (0.59–0.84) ^b^	<0.001	82/191 (42.9%)	0.47 (0.40–0.56) ^b^	<0.001
	6–40 years	39/399 (9.8%)	0.96 (0.93–0.99) ^b^	0.004	33/470 (7.0%)	0.91 (0.87–0.95) ^b^	<0.001	37/494 (7.5%)	0.97 (0.93–1.01) ^b^	0.23
Gender	Male	43/259 (16.6%)	1.0 (reference)	-	42/305 (13.8%)	1.0 (reference)	-	54/290 (18.6%)	1.0 (reference)	-
	Female	67/323 (20.7%)	1.50 (0.99–2.27)	0.05	47/376 (12.5%)	1.12 (0.70–1.79)	0.64	65/395 (16.5%)	0.90 (0.60–1.33)	0.59
Slept under a LLTIN the prior evening	No	70/356 (19.7%)	1.0 (reference)	-	58/480 (12.1%)	1.0 (reference)	-	45/335 (13.4%)	1.0 (reference)	-
	Yes	40/226 (17.7%)	0.55 (0.31–0.97)	0.04	31/201 (15.4%)	1.01 (0.60–1.70)	0.98	74/350 (21.1%)	1.45 (0.89–2.36)	0.14
Wealth index tertiles	Lowest	36/200 (18.0%)	1.0 (reference)	-	36/232 (15.5%)	1.0 (reference)	-	53/230 (23.0%)	1.0 (reference)	-
	Middle	38/192 (19.8%)	1.28 (0.72–2.27)	0.39	27/223 (12.1%)	0.77 (0.39–1.51)	0.45	31/232 (13.4%)	0.63 (0.34–1.16)	0.14
	Highest	36/190 (19.0%)	1.31 (0.71–2.42)	0.39	26/226 (11.5%)	0.80 (0.42–1.53)	0.50	35/223 (15.7%)	0.97 (0.54–1.73)	0.92
Results of blood slide	Negative	95/511 (18.6%)	1.0 (reference)	-	70/594 (11.8%)	1.0 (reference)	-	46/354 (13.0%)	1.0 (reference)	-
	Positive	15/71 (21.1%)	1.59 (0.79–3.17)	0.19	19/87 (21.8%)	2.46 (1.45–4.19)	0.001	73/331 (22.1%)	3.05 (1.92–4.86)	<0.001

^a^ Odds ratio controlling for repeated measures in the same household

^b^ Odds ratio expressed per 1 year increase in age

### Parasitemia

**Fig 3:**
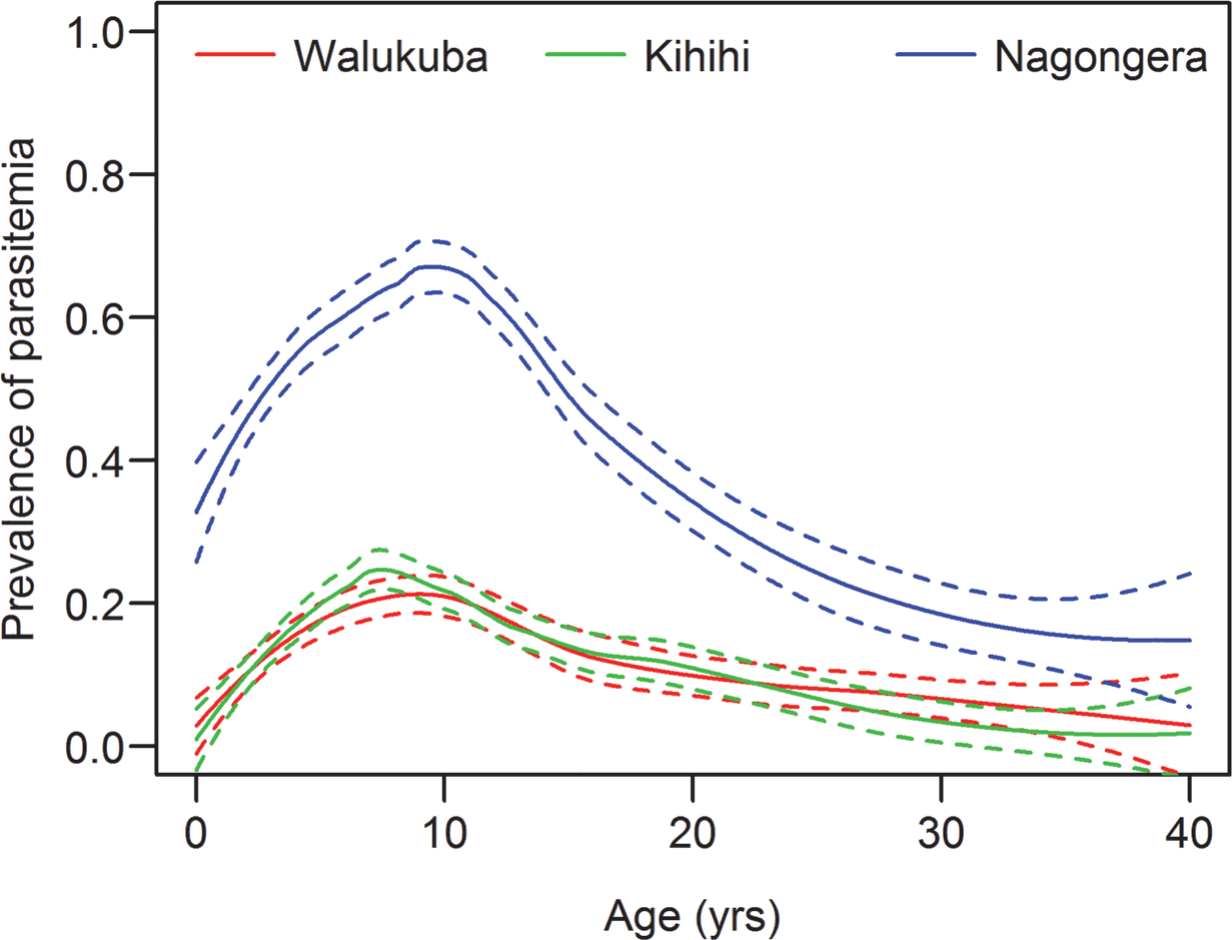
Prevalence of parasitemia (blood slide positive) by age, in three different epidemiological settings in Uganda.

The prevalence of asexual parasitemia was significantly higher in Nagongera than the other two sites (p<0.001 for both comparisons), but was comparable in Walukuba and Kihihi, despite the difference in aEIR in the two sites “Table 2”. A similar pattern was seen for participants of all ages and children aged 2–10 years. Parasite prevalence increased with age, peaking by 11 years, and declining thereafter in all sites (Fig. 3). In the adjusted analysis, age was the only factor significantly associated with parasite prevalence “Table 4”. Although the parasite prevalence was substantially higher in Nagongera compared to the other two sites across all ages, the relationship between age and parasitemia was remarkably similar across all three sites. Increasing age was significantly associated with an increased odds of parasitemia up to 11 years of age (ORs ranging from 1.07 to 1.13 per 1 year increase in age) and then from 12–40 years increasing age was significantly associated with a decreased odds of parasitemia (ORs ranging from 0.89 to 0.92 per 1 year increase in age). Other factors, including gender, ITN use, and socioeconomic status were not significantly associated with parasitemia.

**Table 4:** Factors associated with asexual parasitemia.

Risk factor	Category	Walukuba			Kihihi			Nagongera		
		Parasite prevalence	Adjusted OR^a^ (95% CI)	p-value	Parasite prevalence	Adjusted OR^a^ (95% CI)	p-value	Parasite prevalence	Adjusted OR^a^ (95% CI)	p-value
Age	≤ 11 years	46/314 (14.7%)	1.13 (1.04–1.21)^b^	0.002	58/362 (16.0%)	1.10 (1.02–1.18)^b^	0.009	235/411 (57.2%)	1.07 (1.01–1.13)^b^	0.03
	12–40 years	25/268 (9.3%)	0.92 (0.87–0.97) ^b^	0.001	29/320 (9.1%)	0.89 (0.85–0.94) ^b^	<0.001	96/274 (35.0%)	0.90 (0.87–0.93) ^b^	<0.001
Gender	Male	38/259 (14.7%)	1.0 (reference)	-	44/305 (14.4%)	1.0 (reference)	-	153/290 (52.8%)	1.0 (reference)	-
	Female	33/323 (10.2%)	0.65 (0.39–1.09)	0.10	43/377 (11.4%)	0.85 (0.53–1.37)	0.50	178/395 (45.1%)	0.99 (0.73–1.35)	0.96
Slept under a LLTIN the prior evening	No	40/356 (11.2%)	1.0 (reference)	-	68/481 (14.1%)	1.0 (reference)	-	183/335 (53.6%)	1.0 (reference)	-
	Yes	31/226 (13.7%)	1.52 (0.85–2.72)	0.15	19/201 (9.5%)	0.74 (0.36–1.54)	0.42	148/350 (42.3%)	0.80 (0.53–1.19)	0.27
Wealth index tertiles	Lowest	24/200 (12.0%)	1.0 (reference)	-	22/232 (9.5%)	1.0 (reference)	-	102/230 (44.4%)	1.0 (reference)	-
	Middle	28/192 (14.6%)	1.10 (0.60–2.03)	0.76	34/223 (15.3%)	1.42 (0.71–2.83)	0.32	128/232 (55.2%)	1.48 (0.94–2.33)	0.09
	Highest	19/190 (10.0%)	0.60 (0.28–1.25)	0.17	31/227 (13.7%)	1.12 (0.53–2.38)	0.77	101/223 (45.3%)	0.89 (0.54–1.48)	0.66

^a^ Odds ratio controlling for repeated measures in the same household

^b^ Odds ratio expressed per 1 year increase in age

### AMA-1 antibodies

**Fig 4:**
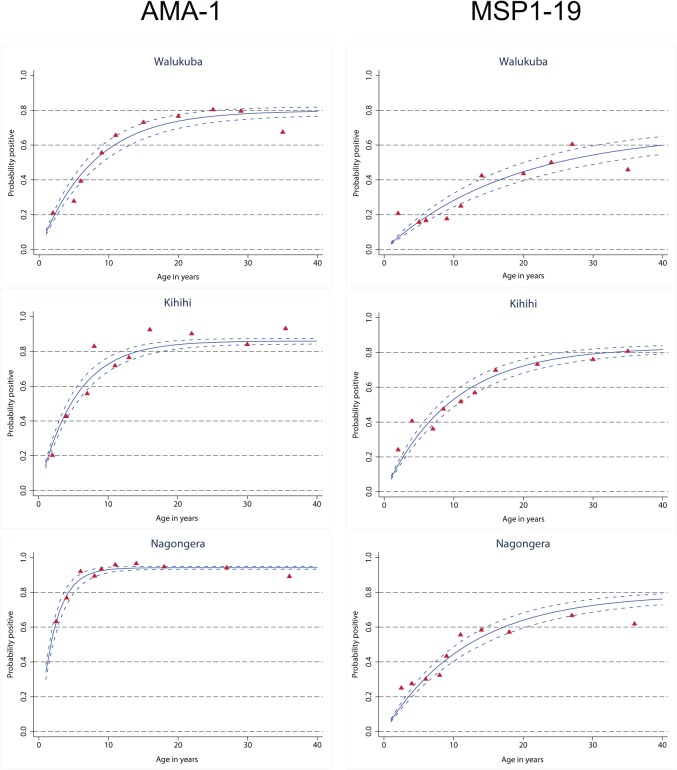
Seroconversion to AMA-1and MSP-1_19_ by age, in three different epidemiological settings in Uganda.

The antibody response to AMA-1, whether evaluated as seroprevalence, titer, or seroconversion rate, increased with increasing transmission intensity “Table 2”. Seroprevalences to AMA-1 were 53.3% in Walukuba, 63.0% in Kihihi and 83.7% in Nagongera (p<0.001 for all comparisons). AMA-1 seroconversion rates were 0.10, 0.17, and 0.42, which, based on prior calibration, corresponded to calculated aEIRs of 3, 9, and 121 for Walukuba, Kihihi, and Nagongera, respectively. Peak seroprevalences were proportionate to the level of transmission intensity and the age at which the peak was reached was inversely proportional to transmission intensity (Fig. 4). In the adjusted analysis, increasing age was associated with increased odds of seropositivity to AMA-1 up to 10 years of age at all three sites with the strength of this association increasing with increasing transmission intensity “Table 5”. In Nagongera, seropositivity to AMA-1 peaked by 10 years of age but continued to increase significantly from 11–20 years of age in Walukuba and Kihihi. Parasitemia was strongly associated with AMA-1 seropositivity at all sites “Table 5”. In Walukuba only, gender and socioeconomic status were also significantly associated with AMA-1 seropositivity, with significantly higher odds for females, and significantly lower odds for those participants of highest socioeconomic status.

**Table 5:** Factors associated with seropositivity to AMA-1.

Risk factor	Category	Walukuba			Kihihi			Nagongera		
		Seropositive	Adjusted OR^a^ (95% CI)	p-value	Seropositive	Adjusted OR^a^ (95% CI)	p-value	Seropositive	Adjusted OR^a^ (95% CI)	p-value
Age	≤ 10 years	100/299 (33.4%)	1.25 (1.15–1.36)^b^	<0.001	137/341 (40.2%)	1.39 (1.28–1.51)^b^	<0.001	274/366 (74.9%)	1.47 (1.31–1.66)^b^	<0.001
	11–20 years	85/117 (72.7%)	1.11 (1.03–1.20)^b^	0.007	155/185 (83.8%)	1.11 (1.01–1.22)^b^	0.03	173/181 (95.6%)	1.00 (0.86–1.16)^b^	0.99
	21–40 years	124/164 (75.6%)	0.96 (0.91–1.02) ^b^	0.21	136/153 (88.9%)	1.00 (0.94–1.06) ^b^	0.94	118/128 (92.2%)	0.96 (0.87–1.05) ^b^	0.35
Gender	Male	118/259 (45.6%)	1.0 (reference)	-	188/304 (61.8%)	1.0 (reference)	-	234/287 (81.5%)	1.0 (reference)	-
	Female	191/321 (59.5%)	1.86 (1.25–2.77)	0.002	240/375 (64.0%)	0.92 (0.65–1.30)	0.64	331/388 (85.3%)	1.40 (0.89–2.18)	0.14
Slept under a LLTIN the prior evening	No	193/354 (54.5%)	1.0 (reference)	-	311/481 (64.7%)	1.0 (reference)	-	292/330 (88.5%)	1.0 (reference)	-
	Yes	116/226 (51.3%)	1.11 (0.73–1.70)	0.63	117/198 (59.1%)	1.21 (0.73–2.00)	0.46	273/345 (79.1%)	0.74 (0.41–1.33)	0.31
Wealth index quartiles	Lowest	120/198 (60.6%)	1.0 (reference)	-	144/230 (62.6%)	1.0 (reference)	-	183/228 (80.3%)	1.0 (reference)	-
	Middle	101/192 (52.6%)	0.74 (0.42–1.29)	0.29	139/222 (62.6%)	0.88 (0.51–1.53)	0.65	200/230 (87.0%)	0.97 (0.50–1.91)	0.94
	Highest	88/190 (46.3%)	0.57 (0.33–0.97)	0.04	145/227 (63.9%)	0.76 (0.42–1.36)	0.35	182/217 (83.9%)	0.76 (0.39–1.48)	0.42
Results of blood slide	Negative	255/509 (50.1%)	1.0 (reference)	-	360/593 (60.7%)	1.0 (reference)	-	268/348 (77.0%)	1.0 (reference)	-
	Positive	54/71 (76.1%)	3.88 (2.03–7.43)	<0.001	68/86 (79.1%)	2.73 (1.44–5.18)	0.002	297/327 (90.8%)	3.39 (1.84–6.25)	<0.001

^a^ Odds ratio controlling for repeated measures in the same household

^b^ Odds ratio expressed per 1 year increase in age

### MSP-1_19_ antibodies

Seroprevalences of antibodies to MSP-1_19_, median MSP-1_19_ antibody titers, and seroconversion rates were lower than with AMA-1 and demonstrated different patterns across the three sites “Table 2”. Seropositivity to MSP-1_19_ was highest in Kihihi, and was significantly lower in Walukuba (31.8%), than in Kihihi (50.7%, p<0.001) and Nagongera (45.7%, p<0.001). MSP-1_19_ seroconversion rates were 0.036, 0.089, and 0.064 for Walukuba, Kihihi, and Nagongera, respectively, which corresponded to calculated aEIRs of 2 in Walukuba and 11 in Kihihi, but only 6 in Nagongera, much lower than the aEIR actually observed (125) [30]. In the adjusted analysis, there was an inconsistent relationship between age and seropositivity to MSP-1_19_ up to 3 years of age across the 3 sites “Table 6”. From 4–20 years of age there was a consistent and significant relationship between increasing age and increasing seropositivity to MSP-1_19_ and no significant relationship from 21–40 years of age across the 3 sites. Only in Walukuba was parasitemia significantly associated with seropositivity to MSP-1_19_ “Table 6”.

**Table 6:** Factors associated with seropositivity to MSP-1_19_.

Risk factor	Category	Walukuba			Kihihi			Nagongera		
		Seropositive	Adjusted OR^a^ (95% CI)	p-value	Seropositive	Adjusted OR^a^ (95% CI)	p-value	Seropositive	Adjusted OR^a^ (95% CI)	p-value
Age	≤ 3 years	25/119 (21.0%)	0.78 (0.60–1.03)^b^	0.08	29/141 (20.6%)	1.23 (0.94–1.62)^b^	0.13	42/121 (34.7%)	0.64 (0.48–0.84)^b^	0.002
	4–20 years	74/272 (27.2%)	1.12 (1.07–1.17)^b^	<0.001	197/385 (51.2%)	1.13 (1.08–1.17)^b^	<0.001	187/429 (43.6%)	1.12 (1.08–1.16)^b^	<0.001
	21–40 years	74/153 (48.4%)	1.00 (0.96–1.06) ^b^	0.88	118/152 (77.6%)	1.01 (0.97–1.05) ^b^	0.59	81/129 (62.8%)	0.98 (0.94–1.02) ^b^	0.33
Gender	Male	69/241 (28.6%)	1.0 (reference)	-	149/304 (49.0%)	1.0 (reference)	-	119/288 (41.3%)	1.0 (reference)	-
	Female	104/303 (34.3%)	1.17 (0.76–1.79)	0.49	195/374 (52.1%)	0.99 (0.73–1.34)	0.94	191/391 (48.9%)	1.18 (0.83–1.68)	0.37
Slept under a LLTIN the prior evening	No	111/335 (33.1%)	1.0 (reference)	-	244/479 (50.9%)	1.0 (reference)	-	161/332 (48.5%)	1.0 (reference)	-
	Yes	62/209 (29.7%)	0.88 (0.57–1.37)	0.58	100/199 (50.3%)	1.09 (0.69–1.70)	0.72	149/347 (42.9%)	0.83 (0.59–1.18)	0.30
Wealth index tertiles	Lowest	68/183 (37.2%)	1.0 (reference)	-	114/230 (49.6%)	1.0 (reference)	-	88/229 (38.4%)	1.0 (reference)	-
	Middle	55/184 (29.9%)	0.77 (0.47–1.26)	0.29	113/221 (51.1%)	1.12 (0.67–1.86)	0.67	116/231 (50.2%)	1.64 (1.11–2.41)	0.01
	Highest	50/177 (28.3%)	0.77 (0.48–1.22)	0.26	117/227 (51.5%)	1.08 (0.62–1.86)	0.79	106/219 (48.4%)	1.48 (0.97–2.26)	0.07
Results of blood slide	Negative	146/477 (30.6%)	1.0 (reference)	-	294/592 (49.7%)	1.0 (reference)	-	172/350 (49.1%)	1.0 (reference)	-
	Positive	27/67 (40.3%)	2.11 (1.16–3.81)	0.01	50/86 (58.1%)	1.27 (0.74–2.17)	0.39	138/329 (42.0%)	0.93 (0.66–1.31)	0.67

^a^ Odds ratio controlling for repeated measures in the same household

^b^ Odds ratio expressed per 1 year increase in age

## Discussion

In this study, we evaluated the prevalence of key malaria indicators and associated factors in three different epidemiological settings in Uganda. The prevalence of anemia was predominant in the youngest children at all sites and did not correlate well with the level of transmission intensity. Severe anemia was uncommon at all sites. The prevalence of parasitemia was highest in Nagongera, the highest transmission site, but results were similar in Walukuba and Kihihi, despite an almost ten-fold difference in aEIR. The relationship between age and parasite prevalence was similar at all three sites, increasing with age until peaking by 11 years and then decreasing with age. Seroprevalence and seroconversion rates of antibodies to AMA-1 appeared to best reflect differences in transmission intensity between the three sites. In contrast, seropositivity of antibodies to MSP-1_19_ did not follow a consistent pattern with age, and the seroconversion rate was unexpectedly low in Nagongera.

Despite the recent increase in donor financing and focus on scaling-up of available malaria control interventions, including ITNs, IRS, IPTp, and treatment with ACTs, coverage levels remain far below targets in Uganda. Although ownership and usage of ITNs has increased substantially in Uganda in the past decade, and ITN distribution has been extended beyond the traditional target groups of children under-five and pregnant women with the recent shift in policy to universal coverage, we found that ≤ 35% of households in the 3 study sites own enough ITNs to cover the household population (one ITN per two residents). Fortunately, Uganda is currently undertaking a national ITN distribution campaign aiming to achieve universal net coverage for the whole country. The same is true for IRS, which was reinstated as a malaria control measure in Uganda in 2006, after a hiatus of about 40 years. Supported by the US President’s Malaria Initiative and other partners, Uganda’s IRS strategy has emphasized implementation in epidemic-prone areas, high-risk situations, such as camps for internally displaced persons or refugees, and more recently, high-transmission areas [32]. But resources for IRS are limited and only a few districts in Uganda benefit from this control strategy. Similarly, coverage of IPTp remains low. We found that the proportion of mothers who received ≥ 2 doses of SP for IPTp during their last pregnancy ranged from 35–53% in the 3 sites; this is below targets, but higher than the estimate of national IPTp coverage (25%) measured in the 2011 DHS survey. On a more positive note, the majority (≥ 75%) of febrile children under-five received ACTs for antimalarial treatment, consistent with results from recent national surveys, suggesting increased access and availability of ACTs in Uganda.

Although the etiology of anemia in tropical areas is multifactorial, *P*. *falciparum* malaria is commonly associated with anemia in children living in holoendemic malaria areas [21,33–35]. We found that anemia was age-dependent at the 3 sites, as reported in other studies [36]. However, the prevalence of anemia did not correlate with the transmission intensity of the sites, and in fact, was highest in Walukuba, the site with lowest transmission. Reasons for this finding are unclear although schistosomasis or hookworm infection may have contributed. There is a complex interplay between anemia, malaria parasitemia, red blood cell disorders, intestinal helminths and malnutrition, however available data seems to suggest that malaria parasitemia is the significant risk factor for anaemia [37,38]. Parasitemia was an independent predictor of anemia at the higher transmission sites of Kihihi and Nagongera, suggesting that malaria plays a role in the burden of anemia in higher transmission settings. This finding is supported by prior studies demonstrating that children living in areas with very high malaria transmission carry a markedly higher burden of anemia and febrile malaria episodes [39,40]. However, we found that severe anemia (hemoglobin < 8.0 g/dL) was uncommon, even in Nagongera, the highest transmission site. In Uganda, the prevalence of anemia, including moderate to severe anemia, in children under-five has declined steadily since 2000, despite the persisting burden of malaria in the country. Although childhood anemia remains an important metric of overall child health, it is possible that malaria may no longer be the primary contributor to anemia in Ugandan children in all settings, raising the question of whether anemia should continue to be used as a metric of malaria-specific disease burden.

Parasite prevalence, particularly in children aged 2–10 years, is a commonly used measure of malaria transmission intensity and disease burden across a range of endemicities [22,23]. However, parasite prevalence is affected by seasonal variation in transmission levels, survey timing, and peak transmission seasons, and may be best considered a short-term measure of malaria [24]. In addition, parasite prevalence has a complex relationship with age and may be influenced by host immunity and use of antimalarial drugs. We found that prevalence of asexual parasitemia was significantly higher in Nagongera, but was comparable in Walukuba and Kihihi, despite the difference in aEIR in the two sites, suggesting that parasite prevalence did not reflect the difference in transmission at the two lower intensity sites. Furthermore, we found that compared to previous surveys parasite prevalence appears to be increasing in Nagongera, decreasing in Walukuba, and is relatively unchanged in Kihihi. Measuring malaria parasite prevalence over time is challenging because malaria transmission dynamics are sensitive to climatic variability [41] and are heterogeneous over small areas [42], complicating interpretation of trends. Fluctuations in climate patterns over several years may also contribute to similar fluctuations in parasite prevalence levels (inter-annual variation) which could mask successes or lapses in malaria control efforts. Thus, although parasite prevalence is arguably the most direct measure of malaria burden, it has several limitations and may not be able to accurate classify the full spectrum of transmission intensity or track temporal changes.

Serological markers are useful adjunct measures of transmission as antibodies can persist for months or years after infection, thereby smoothing out the effects of seasonal or unstable malaria transmission [43] and represent cumulative exposure to infection. At our study sites, antibodies to AMA-1 consistently increased with increasing aEIR with a good correlation between seroconversion rates and measured aEIR. AMA-1 seropositivity increased with age, reaching a higher peak at a younger age as the level of transmission intensity increased. These results support the utility of the prevalence, magnitude and rate of acquisition of antibodies to AMA-1 as a reliable marker of the level of transmission as has been observed previously [22,30]. However, in this setting, antibody responses to MSP-1_19_ did not follow the same pattern; antibody prevalence decreased in the first 5 years of life and the seroconversion rate was surprisingly low for the high transmission site of Nagongera. The former may be due to a combination of short-term fluctuations in transmission and high density parasite infections in some young children. Antibody responses to MSP-1_19_ have recently been shown to be more affected by seasonal changes in transmission [44]. The unexpectedly low seroconversion rate to MSP-1_19_ in Nagongera is consistent with observations in other hyperendemic settings in Uganda and elsewhere [45]. The reasons for this are unclear but a possible explanation may be down-regulation of the immune response at very high transmission levels.

This study was conducted in areas with varying malaria transmission, and the findings may apply in areas of similar transmission patterns. The concurrent measurement of aEIR, parasite prevalence and serology enabled us to compare malariometric indices across different endemicity settings. However, studies were conducted at only 3 sites and we could not assess the trends nor draw firm conclusions on causality because of the cross sectional design. Nonetheless, such surveys are important for tracking temporal trends of malariometric indices and could be used to measure the impact of control interventions.

In summary, we found that coverage of malaria control interventions remains below targets in these 3 sites in Uganda. Although anemia and parasite prevalence are widely used indicators of the burden of malaria, we found that they did not directly correlate with transmission intensity in our study sites. Seroprevalence of AMA-1 was a better marker of infection risk and disease burden and may be a better indicator than anemia or parasitemia for monitoring and evaluation of trends in malaria burden and the impact of malaria control activities.

## Supporting Information

S1 Protocol Study protocol. (DOC)
