# Supplementary material for: Factors Associated with Malaria Parasitemia, Anemia and Serological Responses in a Spectrum of Epidemiological Settings in Uganda
Source: PLoS One. 2015 Mar 13;10(3):e0118901. doi: 10.1371/journal.pone.0118901 (PMC4358889; doi:10.1371/journal.pone.0118901)
Supplement: S1 Protocol — (DOC) [file pone.0118901.s001.doc]

**“Program for resistance, immunology, surveIllance, and modelling of malaria” (PRISM)**

**Study Title: Surveillance of malaria morbidity and coverage of key control interventions: Cross-sectional surveys in communities and schools in three different epidemiological settings in Uganda**

**DMID Protocol Number: 10-0062**

**Sponsored by: National Institute of Allergy and Infectious Diseases (NIAID)**

**DMID Funding Mechanism: NIH/NIAID U19AI089674**

**Principal Investigator: Sarah G. Staedke, MD, PhD**

**DMID Program officer: Malla Rao, DrPH, M Engg**

**Protocol Version 1.0, 04 April 2011**

**Statement of Compliance**

The study will be carried out in accordance with Good Clinical Practice (GCP) as required by the following:

- US Code of Federal Regulations applicable to Clinical Studies (45 CFR)
- *Ugandan Ethics Committee* (or whatever constitutes the national regulatory body)
- Completion of Human Subjects Protection Training
- NIH/NIAID Clinical Terms of Award

**SIGNATURE PAGE**

The signature below constitutes the approval of this protocol and the attachments, and provides the necessary assurances that this study will be conducted according to all stipulations of the protocol, including all statements regarding confidentiality, and according to local legal and regulatory requirements and applicable US federal regulations and ICH guidelines.

Site Investigator:

Signed: ______________________________________________

*Name*

*Title*

## TABLE OF CONTENTS

[TABLE OF CONTENTS 4](#__RefHeading___Toc290051035)

[LIST OF TABLES. 6](#__RefHeading___Toc290051036)

[LIST OF FIGURES. 6](#__RefHeading___Toc290051037)

[DEFINITIONS 7](#__RefHeading___Toc290051038)

[INVESTIGATORS 8](#__RefHeading___Toc290051039)

[COLLABORATING INSTITUTIONS 9](#__RefHeading___Toc290051040)

[INSTITUTIONAL REVIEW BOARDS 10](#__RefHeading___Toc290051041)

[STUDY SUMMARY 11](#__RefHeading___Toc290051042)

[1.0 BACKGROUND 13](#__RefHeading___Toc290051043)

[1.1 Burden of Malaria in Uganda 13](#__RefHeading___Toc290051044)

[1.2 Malaria control in Uganda 13](#__RefHeading___Toc290051045)

[1.3 Surveillance of malaria burden in Uganda 14](#__RefHeading___Toc290051046)

[1.4. Surveillance of malaria specific mortality. 15](#__RefHeading___Toc290051047)

[2.0 RATIONALE 16](#__RefHeading___Toc290051048)

[3.0 OBJECTIVES 16](#__RefHeading___Toc290051049)

[3.1 General objective: 16](#__RefHeading___Toc290051050)

[3.2 Specific objectives: 16](#__RefHeading___Toc290051051)

[4.0 STUDY METHODS 16](#__RefHeading___Toc290051052)

[4.1 Overall study design 16](#__RefHeading___Toc290051053)

[4.2 Study area 17](#__RefHeading___Toc290051054)

[5.0 COMMUNITY SURVEYS 19](#__RefHeading___Toc290051055)

[5.1 Household survey 20](#__RefHeading___Toc290051056)

[5.2 Women’s survey. 21](#__RefHeading___Toc290051057)

[5.3 Clinical survey 22](#__RefHeading___Toc290051058)

[6.0 SCHOOL SURVEYS 23](#__RefHeading___Toc290051059)

[6.1 Overview. 23](#__RefHeading___Toc290051060)

[6.2 Selection criteria 23](#__RefHeading___Toc290051061)

[6.3. Initial recruitment and consent 24](#__RefHeading___Toc290051062)

[6.4 Screening and enrollment of schoolchildren 24](#__RefHeading___Toc290051063)

[6.5 Clinical management and referral. 24](#__RefHeading___Toc290051064)

[6.6 GPS data collection. 24](#__RefHeading___Toc290051065)

[7.0 LABORATORY TESTING 24](#__RefHeading___Toc290051066)

[7.1. Microscopy 24](#__RefHeading___Toc290051067)

[7.2. Measurement of hemoglobin 25](#__RefHeading___Toc290051068)

[7.3. Rapid diagnostic tests 25](#__RefHeading___Toc290051069)

[7.4. Filter paper sample collection 26](#__RefHeading___Toc290051070)

[7.5. Molecular and immunology studies 26](#__RefHeading___Toc290051071)

[8.0 STATISTICAL ISSUES 26](#__RefHeading___Toc290051072)

[8.1 Outcome measures 26](#__RefHeading___Toc290051074)

[8.2 Analytical plan and sampe size/power calculations 27](#__RefHeading___Toc290051075)

[9.0 DATA MANAGEMENT 28](#__RefHeading___Toc290051076)

[10.0 ETHICAL CONSIDERATIONS 29](#__RefHeading___Toc290051077)

[10.1 Institutional Review Boards 29](#__RefHeading___Toc290051078)

[10.2 Informed consent procedures 29](#__RefHeading___Toc290051079)

[10.3 Confidentiality of respondents’ information 29](#__RefHeading___Toc290051080)

[10.4 Potential benefits to participants 30](#__RefHeading___Toc290051081)

[10.5 Potential risks to participants 30](#__RefHeading___Toc290051082)

[10.6 Potential risks to study staff 30](#__RefHeading___Toc290051083)

[10.7 Reimbursement and incentives 30](#__RefHeading___Toc290051084)

[REFERENCES. 31](#__RefHeading___Toc290051085)

[Appendices. 33](#__RefHeading___Toc290051086)

#

# **LIST OF TABLES.**

[Table 1. Characteristics of selected districts 18](#__RefHeading___Toc289798812)

# LIST OF FIGURES.

[**Figure 1. Study sites 19**](#__RefHeading___Toc289798840)

# DEFINITIONS

**Dwelling unit:** is a room or group of rooms occupied by one or more households. It may be distinguished from the next dwelling unit by a separate entrance.

**Household:** consists of a person or group of persons, related or unrelated, who live together in the same dwelling unit, who acknowledge one adult male or female as the head of household, who share the same meals and living arrangements, and are considered as one unit.

**Structure:** is a free-standing building, for a residential or commercial purpose. It may have one or more rooms in which people live; it may be an apartment building, a house, or a thatched hut, for instance.

**Verbal autopsy:** A verbal autopsy is a method of determining the cause of a death based on an interview with next of kin or other caregivers, and involves a questionnaire, method of interpreting the questionnaire results, and a coding scheme.

# INVESTIGATORS

**Sarah Staedke, MD, PhD**

Role in project: Principal Investigator

Clinical Senior Lecturer, London School of Hygiene and Tropical Medicine, London, UK

Co-director, Uganda Malaria Surveillance Project, Kampala, Uganda

Email: [sarah.staedke@lshtm.ac.uk](mailto:sarah.staedke@lshtm.ac.uk)

**Moses Kamya, MBChB, MPH, PhD**

Role in project: Co-Investigator

Professor, Department of Medicine, Makerere University, Kampala, Uganda

Director, Infectious Disease Research Collaboration / Uganda Malaria Surveillance Project, Kampala

Email: [mkamya@infocom.co.ug](mailto:mkamya@infocom.co.ug)

**Grant Dorsey, MD, PhD**

Role in project: Co-investigator

Associate Professor, Department of Medicine, University of California, San Francisco

Co-investigator, Uganda Malaria Surveillance Project, Kampala, Uganda

Email: [gdorsey@medsfgh.ucsf.edu](mailto:gdorsey@medsfgh.ucsf.edu)

**Yeka Adoke MBchB, MPH**

Role in the project: Co-investigator

Epidemiologist, Uganda Malaria Surveillance Project, Kampala, Uganda

Email: yadoke@yahoo.com

**Ann Gasasira MBChB, MSc, PhD**

Role in the project: Co-investigator

Epidemiologist, Uganda Malaria Surveillance Project, Kampala, Uganda

Email: agasasira@gmail.com

**Arthur Mpimbaza MBChB, MMed**

Role in the project: Co-investigator

Pediatrician, Uganda Malaria Surveillance Project, Kampala, Uganda

Email: arthurwakg@yahoo.com

**Joaniter Nankabirwa MBChB, MSc**

Role in the project: Co-investigator

Epidemiologist, Uganda Malaria Surveillance Project, Kampala, Uganda

Email: [jnankabirwa@yahoo.co.uk](mailto:jnankabirwa@yahoo.co.uk)

**Humphrey Wanzira, MBChB, Msc**

Role in the project: Co-investigator/coordinator

Epidemiologist, Uganda Malaria Surveillance Project, Kampala, Uganda

Email: [wanzirah@yahoo.com](mailto:wanzirah@yahoo.com)

# COLLABORATING INSTITUTIONS

**Uganda Malaria Surveillance Project (UMSP)**

Address: Uganda Malaria Surveillance Project, P.O. Box 7475, Kampala, Uganda

Contact person: Catherine Tugaineyo

Phone number: +256 (0) 414-530692

Fax number: +256 (0) 414-540524

Email: [ctugaineyo@muucsf.org](mailto:ctugaineyo@muucsf.org)

**London School of Hygiene & Tropical Medicine (LSHTM)**

Address: Keppel Street, London, WC1E 7HT, UK

Contact Person: Susan Sheedy

Fax Number: +44 (0)20 7637 4314

Phone Number: +44 (0) 20 7927 2256

Email: [susan.sheedy@lshtm.ac.uk](mailto:susan.sheedy@lshtm.ac.uk)

**University of California, San Francisco, (UCSF)**

Address: San Francisco General Hospital, 1001 Potrero Avenue, Building 30, Room 402/408, San Francisco, CA 94110, USA

Contact Person: Tamara Clark

Fax Number: +1 (415) 648-8425

Phone Number: +1 (415) 206-8790

Email: [tclark@medsfgh.ucsf.edu](mailto:tclark@medsfgh.ucsf.edu)

**Makerere University College of Health Sciences, School of Medicine**

Address: Box 7062, Kampala, Uganda.

Contact Person: Moses Kamya

Phone number: +256 (0) 414-530692

Fax number: +256 (0) 414-540524

Email: [mkamya@infocom.co.ug](mailto:mkamya@infocom.co.ug)

# INSTITUTIONAL REVIEW BOARDS

**Uganda National Council for Science and Technology (UNCST)**

Address: Uganda House, 11th Floor, PO Box 6884, Kampala, Uganda

Contact Person: Julius Ecuru

Phone Number: +256-41-250499

Fax Number: +256-41-234579

**Makerere University School of Medicine Research and Ethics Committee (SOMREC)**

Address: Makerere University, Faculty of Medicine, Office of the Dean, PO Box 7072, Kampala, Uganda

Contact Person: Dr. Charles Ibingira

Phone Number: +256 (0) 414-530020

Fax Number: +256 (0) 414-531091

**University of California, San Francisco, Committee for Human Research**

Address: Office of Research, 3333 California Street Suite 315, San Francisco, CA 94118, USA

Contact Person: Dr. Reese T. Jones

Phone Number: +1 (415) 476-1814

Fax Number: +1 (415) 502-1347

**London School of Hygiene and Tropical Medicine Ethics Committee**

Address: Keppel Street, London, WC1E 7HT, UK

Contact Person: Gemma Howe

Phone Number: +44 (0) 20 7927 2802

Email: Ethics@lshtm.ac.uk

# STUDY SUMMARY

| Title | **Surveillance of malaria morbidity and coverage of key control interventions: Cross-sectional surveys in communities and schools in three different epidemiological settings in Uganda** |
| --- | --- |
| Description | Cross sectional surveys conducted twice a year in communities and primary schools in three different sites in Uganda |
| Study sites | 1. **Tororo:** Nagongera sub-county, a rural area in Tororo district in eastern Uganda, is an area of presumed high malaria transmission intensity 2. **Jinja:** Walukuba sub-county, a peri-urban area in Jinja district in the central region, is an area of presumed medium malaria transmission intensity 3. **Kanungu:** Kihihi sub-county, a rural area in Kanungu district in western Uganda, is an area of presumed low malaria transmission intensity |
| Study objectives | 1. To estimate the prevalence of malaria parasitemia and anemia in three different epidemiological settings 2. To compare estimates of the prevalence of malaria parasitemia and anemia as determined in school surveys with those estimated through ‘gold standard’ community surveys 3. To estimate the all-cause mortality rate in children under five in three different epidemiological settings 4. To estimate the malaria-specific mortality rate among children under five years of age using verbal autopsy procedures in three different epidemiological settings 5. To determine coverage of key malaria control interventions (ITNs, IRS, IPTp and treatment with ACTs) in three different epidemiological settings 6. To measure associations between antimalarial antibodies and estimates of malaria parasite prevalence and anemia in school and community surveys |
| Participants and Sample Size | **Community survey:**  Household survey,:  - randomly sample 200 households per site  - administer a household questionnaire to the head of the household or their designate.  Women’s survey:  - sample all women in the reproductive age group (15 – 49 years) in each selected household.  - administer a women’s questionnaire to all selected women  Clinical survey:  **-** sample all children under fifteen years of age in each selected household  - randomly sample one household member within the age category 15-24 years, 25-34 years, 35-44 years, 45-54 years, and > 55 years.  - collect a fingerprick blood sample from each participant for measurement of hemoglobin, thick and thin blood smear, rapid diagnostic test (RDT) for malaria, and filter paper blood sample  **School survey:**  - randomly select 3 primary schools per site.  - randomly select 100 pupils per school (30 children from each of the classes 2-6) |
| Selection Criteria | **Community survey:**  Household survey,:  Inclusion criteria:  1) Usual resident who is present in the sampled household on the night before the survey,  2) aged 18 years or older (adult)  3) agreement to provide informed consent  Exclusion criterion:  1) No adult resident home on more than 3 occasions  2) Household vacant  3) Dwelling destroyed or not found  Women’s survey:  Inclusion criteria.  1) usual female resident present in the sampled household on the night before the survey  2) age 15 – 49 years  3) agreement to provide informed consent (for adult women 18 years and above)  4) agreement to provide assent to participate in the study (for women aged 15-17 years)  5) agreement of parents or guardians of women aged 15-17 years to provide informed consent  Exclusion criteria:  1) inability to locate on more than three occasions.  Clinical survey:  Inclusion criteria:  1) usual resident who is present in the sampled households on the night before the survey  2) resident under 15 years of age or selected household members aged 15 years or older  3) agreement of parents or guardians of children to provide informed consent  4) agreement of eligible adults to provide informed consent  5) agreement of a child aged 8 years or older to provide assent  Exclusion criterion:  1) inability to locate the child on more than three occasions,  2) inability to locate other eligible respondents on more than three occasions.  **School survey:**  Inclusion criteria  - aged ≥ 6 to < 15 years  - student enrolled at participating school in classes 2 to 6  - student has lived in the sub-county for the last six months  - provision of informed consent to participate in study from parent or guardian  - provision of assent to participate in study by student 8 years or older |
| Study outcomes | **Primary outcome**  - prevalence of anemia and malaria parasitemia  **Secondary outcome**  - coverage of ITNs, IRS, IPTp and treatment with ACTs  - all-cause mortality rate in children under five  - prevalence of antimalarial antibodies |

# 1.0 BACKGROUND

## 1.1 Burden of Malaria in Uganda

Malaria is the leading cause of mortality in children under five years of age in Africa and is responsible for 10% of the overall disease burden, 40% of public health expenditure, 30-50% of inpatient admissions, and up to 50% of outpatient visits (1). In Uganda, malaria is endemic in over 95% of the country, with some of the highest malaria transmission intensities reported in the world (2). According to the World Malaria Report, Uganda ranked 6^th^ in terms of number of malaria cases and 3^rd^ in terms of number of malaria deaths (3). The overall malaria-specific mortality is estimated to be between 70,000 and 100,000 child deaths annually in Uganda, a death toll that far exceeds that of HIV/AIDS (4).

In 90-95% of Uganda, malaria transmission is stable and perennial. Seasonal variation associated with the rainy seasons does occur in different geographic areas of Uganda, with the period following the rains associated with the highest malaria transmission. In the highland areas, there is low and unstable transmission with potential for epidemics (2). In the 2009 Uganda Malaria Indicator Survey (UMIS) the prevalence of malaria parasitemia, assessed based on microscopy, was estimated at 50% in children up to 5 years of age (5). Anemia was also very common, with a hemoglobin <11 g/dl seen in over half of children. Parasite prevalence was high (38-63% by blood smear) in all regions except Kampala (5%), the major urban centre, and in the southwestern region, which includes highland areas (12%). As expected, parasite prevalence was lower in urban areas, with increasing educational levels of mothers, and with increasing wealth.

## 1.2 Malaria control in Uganda

Commitment by the global community to decrease the burden of malaria has recently escalated, with concentrated efforts in sub-Saharan Africa. In 2005, the Roll Back Malaria Partnership and World Health Assembly set a target to reduce the number of malaria cases by 50% between 2000 and the end of 2010 and by 75% by the end of 2015 (6) . In order to meet these targets there has been a rapid scale up of proven interventions, notably distribution of long lasting insecticide treated bed nets (LLITNs), indoor residual spraying (IRS) with insecticides, intermittent preventive therapy in pregnant women (IPTp) and prompt and effective treatment with highly efficacious artemisinin combination therapies (ACT).

With continued support from PMI and the GFATM round 7 grant, large scale efforts are underway to increase the number of households owning one or more ITNs to at least 85% and the number of households owning two or more ITNs to at least 60% by 2010 (7). The 2009 UMIS showed marked increases in ITN usage, with 59% of households owning at least one mosquito net, 47% at least one ITN, and 46% at least one long-lasting ITN (5). In addition to ownership, the use of mosquito nets also increased between 2006 and 2009. In children under age 5 years, the percentage sleeping under a net the night before the survey increased from 22% to 41%, and that sleeping under an ITN increased from 10% to 32%. For women, the percentage sleeping under a net increased from 23% to 42% and that sleeping under an ITN increased from 10% to 33%. Net usage was higher among urban than rural women (5)

The strategy for IRS in Uganda has emphasized implementation in epidemic-prone areas, high transmission settings, and high-risk situations, such as camps for internally displaced persons or refugees (8). Thus, IRS has been applied in both highly endemic and epidemic-prone areas, but coverage has been spotty. In the 2009 MIS, only 6% of households had been sprayed in the previous 12 months, coverage similar to that reported in 2006 (9). Coverage was highest in the Mid-northern region (32% of households). Most recently, attention has moved from epidemic-prone areas to those with very high transmission intensity in central and northern Uganda.

The original policy for IPTp was treatment with SP once during the second and third trimesters for pregnant women. The 2006 UDHS indicated that only 37% of pregnant women received at least one dose and only 16% received two doses of SP, with coverage varying greatly in different parts of the country (9). These numbers improved to 45% and 32%, respectively, in the 2009 UMIS (5). Women in urban areas, with more education, and in higher wealth quintiles were more likely than comparators to utilize IPTp. The IPTp policy has recently been revised to provision of SP at every scheduled antenatal clinic visit after quickening (if at least one month apart), to reflect current WHO guidelines. As a result of these efforts, the percentage of pregnant women receiving two doses of SP is expected to increase to 60% in 2011. Similarly, treatment of fevers with an ACT increased from 1% to 14% in children under 5 over the same period

## 1.3 Surveillance of malaria burden in Uganda

The main source of malaria data in Uganda is routine morbidity case reports. All health facilities are required to provide monthly reports on malaria diagnoses. However, these reports are subject to gross over- or under-estimations of malaria prevalence, as they are generally based on diagnoses without laboratory confirmation and represent only cases presenting to public sector health facilities.

The most robust sampling framework for national malaria surveys are household cluster surveys which collect information on malaria intervention coverage, patterns of antimalarial use, and in some cases, on the prevalence of malaria infection among pregnant women and children under five years.The Uganda Demographic Health Survey (UDHS) provides comprehensive surveys every 5 years as part of a worldwide project. They are based on representative household samples, providing estimates of a range of demographic and health indicators. In Uganda, 4 surveys have been conducted, in 1988-89, 1995, 2000-01 and 2006. Malaria indicators from these surveys include ownership and use of ITNs, use of IRS, coverage of IPTp, and nature of treatment of childhood fevers. In November 2009 the first UMIS was conducted, and collected comprehensive data relevant for assessing core malaria indicators, including population-level coverage of ITNs, IRS, IPTp, and ACTs as well as markers for anemia, parasite prevalence, and infecting parasite species. However, estimating PR among these age groups is not optimal, as pregnant women sequester infections (10) and infection prevalence in very young children is modified by a variety of factors, including presence of maternal antibodies (11). More importantly, national cluster surveys are expensive, time-consuming, and technically complicated to undertake, and sampling is typically powered to provide only national or first-level administrative unit representative estimates of malaria risk and intervention coverage. Such limitations preclude frequent monitoring and evaluation, especially at local levels, hindering decentralized planning and allocation of resources for targeted control. Increasing the frequency of monitoring enables prompt feedback of intervention effectiveness, helping control programs to adapt and improve control strategies. These surveys are unattractive to rely on for routine malaria surveillance in a resource limited setting.

A complementary, inexpensive framework for malaria planning, monitoring and evaluation is offered by school malaria surveys (12-14). The practical advantages of sampling children at school are clear: identification and selection of individuals is simplified, compliance is high, and costs are reduced, since only a fraction of the population is examined. In addition, ministries of education are increasingly developing or upgrading national school databases using geographical information systems (GIS). This allows incorporation of information on school locations and enrolment into a single database, and the use of geo-statistical methods to model risks between schools and across un-sampled schools. There are also important epidemiological reasons for sampling children who attend school. Historically, malaria endemicity was defined on the basis of PR among children aged 2-10 years, and geographical reconnaissance of malaria was recommended in all areas prior to a control program launching into an attack phase of the Global Malaria Elimination Program (15). Recent mathematical models of malaria transmission dynamics indicate that EIR determines both the rate at which PR rises during early childhood and the age at which maximum PR is attained (16). Age-stratified studies in varying transmission settings reveal that PR consistency has a convex relationship with age, with PR rapidly rising among young children, attaining a maximum within the 5-10 year old age class, and declining among adolescents and young adults, thereafter maintaining a relatively stable low value throughout adulthood (17). Such consistency in the relationship between PR and age permits age-standardization of available PR estimates to the 2 to 10 year age range on the basis of catalytic conversion models (17), although estimates are most precise when the majority of the sampled population are within this age range.

The Uganda Malaria Surveillance Project (UMSP) is implementing routine surveillance of outpatient monthly malaria cases, laboratory confirmed suspected malaria cases, the prescription practices of health workers and anti-malarial drug usage at six sites across the country.

## 1.4. Surveillance of malaria specific mortality.

Estimating malaria-specific mortality is challenging (18). Establishing cause-specific mortality rates for malaria and other illness in developing countries is often limited by lack of good quality data on the determinants of death (19). In addition, approximately 80% of deaths occur at home outside the formal health setting and go unnoticed (20). Although improvement of vital registration systems is a long-term goal for many countries, and may help to improve data collection, in the short-term an alternative method for establishing mortality data is needed (21).

Verbal autopsy (VA) is an indirect method of determining cause of death based on an interview with the caretakers of a deceased individual. Information about the presence or absence of specific signs and symptoms, and circumstances preceding the terminal event, are used to ascertain the most likely cause or causes of death. Diagnoses are assigned by physician review of the questionnaire, or using an expert or data-derived algorithm, and are coded by a cause-of-death or mortality classification system (19;22). Coded data are then used to estimate cause-specific mortality fractions. Despite its limitations, such as imperfect sensitivity for determining malaria attributable deaths, VA procedures remain the only viable option for estimating malaria specific deaths rates in communities where alternative sources of accurate data are lacking (23). The World Health Organization has developed standards for harmonization of procedures used to perform VA surveys in such settings (24) (World Health Organization (2007) Verbal autopsy standards: Ascertaining and attributing cause of death. Geneva: World Health Organization).

# 2.0 RATIONALE

The epidemiology of malaria in Africa is in transition, with evidence of declining transmission and disease burden (25-27), a presumed consequence of expanded intervention coverage of insecticide-treated bednets (ITNs), indoor residual spraying (IRS) and effective case management with artemisinin-based combination therapies (ACTs). In Uganda, there has been very little scientific effort to document the transitioning epidemiology using validated metrics of intervention coverage, infection risk, and disease burdens within a robust framework of malaria surveillance. Reliable surveillance is essential for the design, refinement and resource allocation of control programs and to provide assessment of the impact and cost-effectiveness of program activities.

The capacity to conduct high-quality malaria surveillance is currently inadequate in Uganda. Routine facility data collected through the Health Management Information System (HMIS) is subject to the vagaries of incomplete data and inaccurate diagnosis. Furthermore, data on measures of transmission intensity and host-based measures of infection and disease are not collected routinely. There is a lack of understanding of the optimal approaches to routine malaria surveillance in Uganda. We propose to carry out bi annual household (community) and school surveys to estimate malaria morbidity, mortality and coverage of key control interventions at three different transmission sites in Uganda. The proposed studies shall help to establish and validate simplified, reliable, and cost-effective surveillance tools at various malaria transmission settings. This work will inform malaria control efforts in Uganda and provide a model for malaria surveillance in other malaria endemic countries.

# 3.0 OBJECTIVES

## 3.1 General objective:

To estimate indicators of malaria morbidity and coverage of key control interventions in three different epidemiological settings, to help identify optimal strategies for malaria surveillance in Uganda

## 3.2 Specific objectives:

1. To estimate the prevalence of malaria parasitemia and anemia in three different epidemiological settings
2. To compare estimates of the prevalence of malaria parasitemia and anemia as determined in school surveys with those estimated through ‘gold standard’ community surveys
3. To estimate the all-cause mortality rate in children under five in three different epidemiological settings
4. To estimate the malaria-specific mortality rate among children under five of years of age using verbal autopsy procedures in three different epidemiological settings
5. To determine coverage of key malaria control interventions (ITNs, IRS, IPTp and treatment with ACTs) in three different epidemiological settings
6. To measure associations between antimalarial antibodies and estimates of malaria parasite prevalence and anemia in school and community surveys

# 4.0 STUDY METHODS

## 4.1 Overall study design

We propose to conduct cross-sectional surveys in communities and primary schools twice a year for at least 2 years in the 3 different sites. The surveys will be timed to correspond to the high and low malaria transmission seasons, when possible. In the community surveys, 200 households will be recruited from a list randomly generated from a census database at each site. The community surveys will consist of three components: (1) a household survey targeting heads of households, (2) a women’s survey targeting all women of child-bearing age (15-49 years), and (3) clinical surveys of all children under fifteen years of age, and of one household member within the age category 15-24 years, 25-34 years, 35-44 years, 45-54 years, and > 55 years. The clinical surveys will include a fingerprick blood sample for measurement of hemoglobin, thick and thin blood smear, rapid diagnostic test (RDT) for malaria, and filter paper blood sample. The school surveys will be conducted in 3 randomly selected primary schools per site. A total of 100 randomly selected children will be recruited in each school. The school surveys will include only the clinical assessments including measurement of temperature, and a fingerprick blood sample for measurement of hemoglobin, thick and thin blood smear, RDT for malaria, and filter paper blood sample.

##

## 4.2 Study area

The study will be conducted in the sub-counties surrounding UMSP level IV malaria sentinel health centers in Nagongera in Tororo district, Walukuba in Jinja District and Kihihi in Kanungu district. These catchment areas were chosen to represent varied malaria transmission settings in Uganda.

Nagongera is a rural sub-county found in the north-western part of Tororo. The total population of Nagongera sub-county is 37,714 people. Entomology studies conducted in Nagongera in 2001-02 estimated the entomological inoculation rate (EIR) to be 562 infective bites per person per year (2). Nagongera Health Center IV, one of the UMSP’s malaria sentinel health facilities, is the largest public health facility in the sub-county and treats an average of 586 patients per month. Between January 2007 and October 2010, the median monthly age-adjusted slide positivity at the health center was 42% (IQR: 34-44%) (umsp.muucsf.org).

Jinja district is located in the eastern part of the country. Walukuba subcounty is a peri-urban area in the outskirts of Jinja town. The district has an estimated population of 387,600 people, and is an area of medium malaria transmission with an estimated EIR of 6 infective bites per person per year (2). Walukuba health center IV is the largest public health facility in the sub-county and treats an average of 2,514 patients per month (umsp.muucsf.org). Between January 2007 and October 2010 the median monthly age-standardized SPR at Walukuba HC IV was 39% (IQR: 36%- 47%).

Kihihi is a rural sub-county in Kanungu district located in the south western part of Uganda. The district has an estimated population of 204,700 people and is an area of relatively low malaria transmission intensity with an estimated EIR of 6 infective bites per person per year (2). The largest healthcare point in Kihihi sub-county is Kihihi health center IV that also serves as the UMSP malaria sentinel health facility. It provides free health care to the residents of the sub-county and treats an average of 1,742 patients per month (umsp.muucsf.org) with a median monthly age-standardized SPR of 24% (IQR: 18 – 32%) between January 2007 and October 2010.

The local malaria epidemiology highlighting the diversity of malaria ecology at the three study districts is summarized in Table 1.

Table 1. Characteristics of selected districts

| Characteristics | **DISTRICT** | | |
| --- | --- | --- | --- |
|  | Kanungu | **Jinja** | Tororo |
| Demographics ^a^ | | | |
| Population | 204700 | 387600 | 536,900 |
| Urbanization level | 6.3% | 22.1% | 6.5% |
| Number of households | 43500 | 84000 | 112,300 |
| Persons per household | 4.7 | 4.5 | 4.8 |
| Entomology (data collected 2001-2002) | | | |
| Entomologic inoculation rate | 6 | 6 | 562 |
| Predominate vector species | *An. gambiae ss* | *An.gambiae ss* | *An.gambiae ss* |
| Measures of infection (data collected in children 2-9 years of age, 1999) | | | |
| Parasite rate | 43% | 15% | 91% |
| Malaria control interventions | |  |  |
| IRS, year (% targeted coverage) | 2007 (99%) | None | None |
| ITN coverage (< 5 yrs)^d^ | 6.5% | 5.8% | 12.8% |
| > 2 doses of IPTp at ANC visit^d^ | 24.6% | 14.0% | 15.7% |
| Prompt treatment of fever (< 5 yrs) ^d^ | 23.8% | 22.0% | 31.7% |
| ACT coverage^e^ | 81% | 55% | 43% |
| Outpatient level IV health facility data (UMSP data December 2009 – May 2010) | | | |
| Name of health facility | Kihihi | Walukuba | Nagongera |
| Average Number of patients per month | 2117 | 3048 | 1891 |
| Proportion with suspected malaria | 73% | 76% | 73% |
| Slide positivity rate (< 5 yrs) | 46% | 44% | 69% |
| Slide positivity rate (> 5 -15 yrs) | 44% | 48% | 48% |

^a^ Uganda Population and Housing Census, 2002 (Ugandan Bureau of Statistics)

^b^ Data only available from September – November 2008

^d^ Uganda Demographic and Health Survey, 2006

^e^ Proportion of patients with malaria prescribed an ACT at UMSP sentinel health facilities (December 2008-May 2009)

Figure 1. Study sites

**
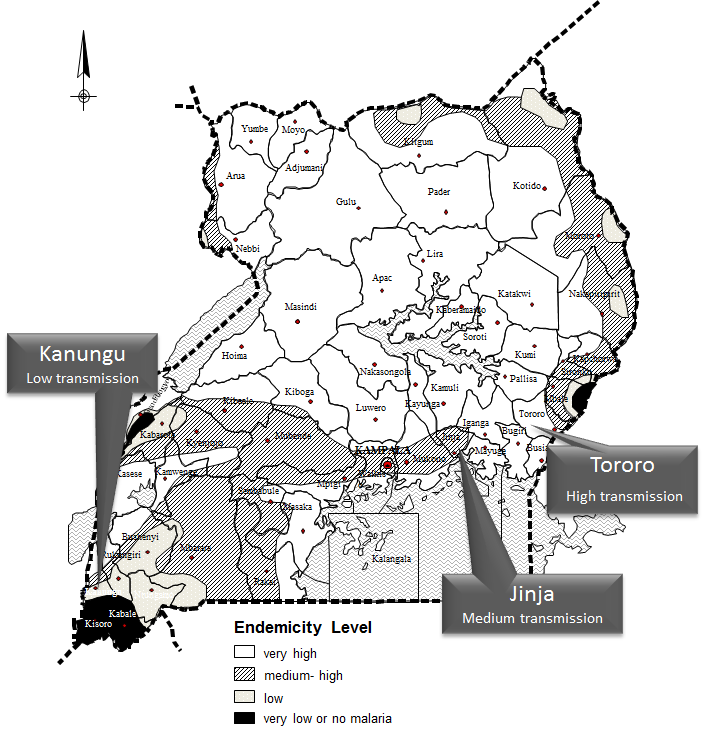
**

##

## 5.0 COMMUNITY SURVEYS

In the community surveys, 200 households will be recruited from a list randomly generated from a census database. The community surveys will consist of three components: (1) a household survey consisting of a questionnaire administered to heads of households, (2) women’s survey consisting of a questionnaire administered to all women of child-bearing age (15-49 years), and (3) clinical surveys consisting of biomarker testing of all children under fifteen years of age, and of one household member within the age category: 15-24 years, 25-34 years, 35-44 years, 45-54 years, and > 55 years.

## 5.1 Household survey

### 5.1.1 Selection of households

A sample of 200 residential households will be selected in each site. In order to create a sampling frame for the selection of participating households, all residential households within each of the three sites have been enumerated and mapped prior to the onset of the surveys as part of a separate protocol. A random sample of households will be selected from each site using computer-generated random numbers to generate a list of households to be approached. Study personnel will conduct door-to-door recruitment to identify those households with at least one adult household resident in sequential order according to the recruitment list. Households without a child of appropriate age will be removed from the recruitment list. Residents not home during the initial contact will be re-visited on at least three other occasions before eliminating them from our sample selection process. New recruitment lists will be randomly generated for each survey using replacement.

### 5.1.2 Screening of respondents.

When a household with at least one household adult resident is identified, study personnel will briefly describe the purpose of the study to the head of the household or any adult household member present at home in the appropriate language (usually Luganda or Lusoga for those in Walukuba; Rukiga or Runyankore for those in Kihihi, and Japadhola and Ateso for those in Nagongera sub-county). The inclusion criteria are: 1) Usual resident who is present in the sampled household on the night before the survey, 2) adult aged 18 years or older, 3) agreement to provide informed consent. The exclusion criterion are: 1) no adult resident home on more than 3 occasions, 2) household vacant, 3) dwelling destroyed or not found

### 5.1.3 Informed consent

Study personnel will conduct the informed consent discussion in the appropriate language and a translator will be used if necessary. The consent form will be available in English and the local languages (Appendix 1). Following the informed consent discussion, the respondent will be asked by the study personnel to sign a written consent form to participate in a research study. If the respondent is unable to read or write, their fingerprint will substitute for a signature, and a signature from a witness to the informed consent procedures will be obtained.

### 5.1.4 Household survey questionnaire

The household questionnaire (appendix 9) will be administered to the head of the household or their designate after obtaining their consent using a hand-held computer (paperless system). The questionnaire is based on the model Malaria Indicator Survey questionnaire developed by the Roll Back Malaria Monitoring and Evaluation Reference Group, as well as other questionnaires from previous surveys conducted in Uganda, including the 2006 Uganda Demographic and Health Survey (UDHS) and the 2009 and 2010 Malaria Indicator Survey.

The Household Questionnaire will obtain information on all residents in the selected households. Some basic information will be collected on the characteristics of each person listed, including age, sex, and relationship to the head of the household. Demographic information from the Household Questionnaire will be used to identify women who are eligible for the individual women interviews and those eligible for the clinical survey. The Household Questionnaire will also be used to collect information on indicators of ownership and use of mosquito bed nets. It will also collect proxy indicators of wealth based on responses of household’s dwelling unit, ownership of various durable goods and land, and household food security.

## 5.2 Women’s survey.

### 5.2.1 Recruitment of participants

The women’s survey targets all women of child-bearing age (15-49 years). The women shall be recruited from every household selected for the household survey.

### 5.2.2 Screening of participants

During the household survey interview, information is obtained on all residents in the selected households. The demographic information from the Household Questionnaire will be used to identify women who are eligible for the individual women interviews. Eligible women will be approached by study personnel who will briefly describe the purpose of the study to them in the appropriate language. The study inclusion criteria is: 1) usual female resident who is present in the sampled household on the night before the survey, 2) age 15 – 49 years, 3) agreement to provide informed consent (for adult women 18 years and above), 4) agreement to provide assent to participate in the study (for women aged 15-17 years), 5) agreement of parents or guardians of women aged 15-17 years to provide informed consent. The exclusion criterion is: 1) inability to locate on more than 3 occasions

### 5.2.3 Informed consent

Study personnel will conduct the informed consent discussion in the appropriate language and a translator will be used if necessary. Consent forms will be available in English and the local languages. Following the informed consent discussion, women aged 18 years and older will be asked by the study personnel to sign a written consent form to participate in a research study (appendix 1). Written assent to participate in the study will also be obtained from women aged 15 – 17 years at the time of the screening (appendix 2). The head of the household or their designate shall provide a second approved consent for women aged 15-17 years to participate in the study (appendix 1). If the respondents are unable to read or write, their fingerprint will substitute for a signature, and a signature from a witness to the informed consent procedures will be obtained.

### 5.2.4 Women’s questionnaire

After obtaining consent from the eligible women, interviewers will administer the women’s questionnaire (appendix 10) using a hand-held computer. Face-to-face personal interviews using a structured standard questionnaire will be administered. The Woman’s Questionnaire will be used to collect information on background characteristics (age, education, etc.); intermittent preventive treatment for malaria; current pregnancy; and antimalarial treatment for children under five with recent fever. It will also collect information on knowledge about malaria and cost of treatment of children under five with fever. In addition, if any of the women has given birth, she will be questioned about the vital statistics of her children (date of birth of each child, whether the child is still alive, and the age of death for any dead child).

### 5.2.5 Verbal autopsy procedures

If during the interview a death of child under five years of age is noted to have occurred during the past 5 years from the date of the interview, a Verbal Autopsy interview will be conducted to ascertain the cause of death (appendix 11 + 12). The mother of the deceased child and any other persons who took care of the child during the illness that resulted in death of the child will be identified. The caretakers will be expected to remember, recollect and give an accurate account of the circumstances leading to the death, and the signs and symptoms of the illness. The interview will be conducted using the Verbal Autopsy questionnaire at the convenience of the caretakers. In the event that the mother/caretakers of the deceased child does not feel comfortable to take part in the interview at that time, an alternative date will be set for the interview and the house re-visited for that purpose.

Completed Verbal Autopsy questionnaires will be photocopied and distributed to two independent physicians. The physicians will first review the questionnaires and assign the cause or causes of death according to their clinical opinion (physician review). Causes of death will be classified as immediate, underlying, and contributory causes, according to ICD-10 guidelines. Discrepancies in diagnoses will be documented and reviewed by a third physician, and a consensus will be sought with the original two physicians. The causes of death will be assigned the appropriate ICD-10 codes by the physicians using a standardized coding list. Deaths attributable to malaria will be considering cases in which malaria was classified as either the underlying or contributory cause of death in different epidemiological settings.

## 5.3 Clinical survey

### 5.3.1 Recruitment of participants

In each selected household, a clinical survey shall be conducted on all children under fifteen years of age, and on one household member within the age category 15-24 years, 25-34 years, 35-44 years, 45-54 years, and > 55 years.

### 5.3.2 Screening of participants

At the end of the household questionnaire, the study personnel will discuss the clinical survey aspect of the study with the head of the household or their designate and adult household members present. Study personnel will briefly describe the purpose of the clinical study in the appropriate language. The inclusion criteria are: 1) Usual residents who are present in the sampled households on the night before the survey, 2) children under 15 years of age or selected household members aged 15 years or older, 3) agreement of parents or guardians of children to provide informed consent, 4) agreement of eligible adults to provide informed consent and 5) agreement of a child aged 8 years or older to provide assent. The exclusion criterion is: 1) inability to locate the child on more than 3 occasions, 2) inability to locate other eligible respondents on more than 3 occasions. All children under 15 years of age will be included for the clinical survey. One person in each of the age category 15-24 years, 25-34 years, 35-44 years, 45-54 years, and > 55 years shall be randomly selected for the clinical survey.

### 5.3.3 Informed consent

Study personnel will conduct the informed consent discussion with the parent(s) or guardian(s) of children and with the other adult respondents. Informed consent will be conducted in the appropriate language and a translator will be used if necessary. Consent forms will be available in English and the local languages. Following the informed consent discussion, parents/guardians will be asked by the study personnel to sign a written consent form for their children to participate in a research study (appendix 3) and a second approved consent form for the future use of biological specimens obtained during the course of the study (appendix 4). If the parent/guardian is unable to read or write, their fingerprint will substitute for a signature, and a signature from a witness to the informed consent procedures will be obtained. Written assent to participate in the study will also be obtained from children aged 8 years and older at the time of screening (appendix 5). Respondents aged 18 years and older will be asked by the study personnel to sign a written consent form to participate in a research study (appendix 3) and a second approved consent form for the future use of biological specimens obtained during the course of the study (appendix 4). If the respondent is unable to read or write, their fingerprint will substitute for a signature, and a signature from a witness to the informed consent procedures will be obtained

### 5.3.4 Clinical survey procedures

The clinical surveys will include a fingerprick blood sample for measurement of hemoglobin, thick and thin blood smear, rapid diagnostic test (RDT) for malaria, and filter paper blood sample. All children under 15 years will have a fingerprick blood sample taken for measurement of hemoglobin, thick and thin blood smear, rapid diagnostic test (RDT) for malaria, and filter paper blood sample. One person randomly selected in each of the age category 15-24 years, 25-34 years, 35-44 years, 45-54 years, and > 55 years shall have a fingerprick blood sample taken for measurement of hemoglobin, thick and thin blood smear, rapid diagnostic test (RDT) for malaria, and filter paper blood sample.

### 5.3.5 Clinical management

Respondents who are tested with an RDT and test positive for malaria shall be offered treatment for malaria in their homes. Respondents with a positive RDT and no evidence of severe malaria will be treated with artemether-lumefantrine, the standard national regimen. Respondents with a positive RDT and evidence of danger signs of severe disease will be referred to the nearest health facility for further evaluation and treatment. Respondents with a hemoglobin level of < 5.0 g/dL will be referred to the nearest health facility for further evaluation and transfusion. Any respondent with other concerning clinical symptoms will also be referred to an appropriate health care facility at the discretion of the study personnel.

## 6.0 SCHOOL SURVEYS

## **6.1 Overview.**

Surveys will be conducted in three schools per site which will be randomly selected from a list of all primary schools in the sub-county generated during an enumeration exercise which has already been completed as part of a separate protocol. The surveys will be done twice a year (once during the rainy season and once during the dry season). New lists of three schools will be randomly generated for each survey using replacement; as a result the same school may participate in the survey more than once.

## 6.2 Selection criteria

Children enrolled in participating schools will be assessed for the following eligibility criteria:

**Inclusion criteria**

1. Aged ≥ 6 to < 15 years
2. Student enrolled at participating school in classes 2 to 6
3. Student has lived in the sub-county for the last six months
4. Provision of informed consent from parent or guardian
5. Provision of assent by student

## 6.3. Initial recruitment and consent

Schools in the participating sub-counties will be selected using simple random sampling from a list of all primary schools in the sub-county generated during the enumeration exercise. Prior to the day of the survey, staff from participating schools will be sensitized about the study and plans for recruitment. A random sample of 150 children aged 6-15 years will be selected from each school using the school register. Thirty children will be randomly selected from each of the classes 2-6 in the selected schools to participate in the study. The parents/guardians of selected children will be invited to the school for a meeting with the study teams, to review the selection criteria for study participation and to gain informed consent. During the meetings, the purpose and procedures of the study will be discussed, informed consent forms will be distributed (appendix 6), and written informed consent will be sought from the parents/guardians. Those parents who do not attend the school meetings will be visited at home and the consent will be sought from their homes. Consent forms will be made available in the local languages and in English. Consent to take part in the research study (appendix 6) and consent for future use of biological specimens (appendix 7) will be sought. Details about the location of the students’ homes will also be obtained from the parent/guardians to facilitate tracing in the event the child is selected for household mapping.

## 6.4 Screening and enrollment of schoolchildren

Clinical evaluation will be conducted at the participating schools. Assessment for eligibility will be done by the study staff, and interviews will be conducted in the appropriate language with the schoolchildren. During the evaluation process, the study personnel will assess for eligibility criteria through conversations with the student. For children fulfilling the eligibility criteria, the study staff will seek assent from the student to participate in the study (appendix 8). The first 20 children from each of the classes 2-6 (100 total) meeting these eligibility criteria will be enrolled into the study. All enrolled children will have basic socio-demographic data including age, sex, weight, and bed net use collected. Tympanic temperature will be measured and recorded for these children. Each child selected will be asked to provide a finger-prick blood sample for testing for malaria parasitemia using an RDT, preparation of thick and thin malaria blood smears, filter paper blood samples and for hemoglobin estimation. All data will be collected using a hand-held computer (appendix 13).

## 6.5 **Clinical management and referral**.

All children with a positive RDT will be treated on site with the nationally recommended age-specific drug dosing regimen by a qualified nurse. Children identified as severely anemic (hemoglobin levels < 5 g/dL) will be referred to the nearest health facility for treatment according to national guidelines

## 6.6 GPS data collection.

Households of a subsample (20%) of the selected children will be mapped using hand-held GPS receivers. These households will be randomly selected from the list of surveyed children. The study team will escort the selected children home; verbal consent will be sought from an adult respondent found at home and GPS readings will be taken from the door of the household, if possible, or from a point that is most representative of the household. At each household, a reading will be taken every five seconds for 2 minutes, and the average values from these readings will be recorded (Easting, Northing, and Altitude) in UTM units. The GPS data collected will be used to estimate the geographical distribution of the students attending the school.

# 7.0 LABORATORY TESTING

## 7.1. Microscopy

Thick and thin blood smears will be prepared in the field for microscopy. New glass slides, frosted at one end, will be used to make the malaria blood smears. Before making the smears, the technician will place a barcode label on the under-side of the glass slides at the frosted end linked to the household questionnaire. At the end of each day, the thin smears will be immersed in absolute methanol for a few seconds in order to prevent auto-fixation of the blood cells. Slides will be kept at the field site protected from excessive heat and light for no longer than 2 weeks. The slides will be kept in a drying box with desiccant and stored in the coolest place possible. The desiccant shall be checked daily to ensure that it is working properly. The blood slides for malaria will be periodically transported to the UMSP Molecular Research Laboratory in Mulago. All specimens will be accompanied by a list of samples generated from the questionnaire and linked to the bar codes of the specimens.

The blood slides for malaria will be read at the UMSP Molecular Research Laboratory in Mulago. Thick and thin blood smears will be stained with 2 percent Giemsa for 30 minutes. Thick blood smears will be evaluated for the presence of parasitemia (asexual forms only) and gametocytes. Parasite and gametocyte densities will be calculated from thick blood smears by counting the number of asexual parasites and gametocytes, respectively, per 200 leukocytes (or per 500, if the count is less than 10 parasites or gametocytes per 200 leukocytes), assuming a leukocyte count of 8,000/μl. A thick blood smear will be considered negative when the examination of X 100 high power fields does not reveal asexual parasites or gametocytes. Thin blood smears will be evaluated to determine parasite species. Thin smears will be read only when the thick smear reading is positive. For quality control, all slides will be read by a second microscopist and a third reviewer will settle any discrepant readings.

Results of blood slides will not be returned to participants. RDT results, however, will be provided to participants on the spot and treatment offered if RDT results are positive, and the participants will be told to seek care at the health facility if illness worsens. It is possible that for some cases, the RDT will be negative, but microscopy will be positive. The most likely reason for an RDT to be negative when microscopy is positive comes from those people with low levels of parasitemia below the level of detection for RDTs but not below the level for microscopy. The risk of developing symptomatic or severe illness is very low from a presumably low parasitemia in an RDT-negative asymptomatic individual. It is worth noting that RDTs are used nationally at the point of care in health facilities, and confirmatory microscopy is usually not performed. Regardless of the RDT results, all participants will be encouraged to seek treatment at health facilities should they become ill following the survey.

## 7.2. Measurement of hemoglobin

Hemoglobin analysis will be carried out on site using a drop of blood collected from a finger-stick (or heel stick). The test will be conducted using a battery-operated portable HemoCue analyzer which produces a result within one minute. Results will be returned verbally and in written form and those who have severe anemia requiring treatment (under 5 g/dl) will be provided with information on local health facilities where treatment can be obtained and they will be urged to go to these health facilities for treatment. Results of the anemia test will be entered into the Household Questionnaire.

## 7.3. Rapid diagnostic tests

Using the same fingerprick made for anemia testing, a drop of blood will be tested immediately using the Paracheck rapid diagnostic test, which tests for *Plasmodium falciparum.* The test includes a loop applicator that comes in a sterile packet. A few microlitres of blood is captured on the applicator and placed on the well of the device. Results are available in 15 minutes. The results will be provided to the respondent in oral and written form and will be recorded on the Household Questionnaire. Those who test positive for malaria will be provided a full course of treatment for malaria according to the national standard malaria treatment guideline in Uganda. They will also be told to go to the nearest health facility immediately if their illness worsens.

## 7.4. Filter paper sample collection

Blood spots will be collected onto prepared filter papers (Whatman no 1, Whatman 3MM; Whatman, Maidstone, UK) in volumes from 1 μl to 45 μl, as per established protocol. Spots will be allowed to dry at ambient temperature and relative humidity overnight and labelled with the individual’s bar codes or ID number on the covering cardboard. Each filter paper will be stored in individual self-sealing plastic bags. These bags will then be transported to a storage facility in boxes with silica gel sachets; each box will be well labelled with the survey details. The blood spots will be used for molecular and immunology studies.

## 7.5. Molecular and immunology studies

DNA will be extracted from filter papers using standard chelex methodology and tested for the presence and speciation of malaria parasites based on nested PCR of cytochrome b (28). Reconstituted filter paper spots will be tested for human malaria-specific IgG antibodies (e.g. anti-MSP-1_19_ anti-AMA-1) by ELISA using standard methodology (29). The prevalence and density of antibody responses will be determined. For antibody prevalence, a cut off above which samples are deemed antibody positive is defined using a mixture model and a pool of malaria naïve European control sera. The sero-conversion rate (SCR or λ) as serological marker of exposure will be estimated by fitting a simple reversible catalytic model to the combined antibody prevalence, stratified into yearly age-groups (29). In the household (community) surveys, the SCR will be determined for each of the three areas separately. The SCR will be related to parasite prevalence and anemia data in the different areas at different time-points. Evidence for changes in transmission intensity will be explored by fitting different forces of infection. In the school surveys a similar approach will be used, estimating an SCR for school-age children. In addition, age-standardized antibody prevalence and density will be calculated.

# STATISTICAL ISSUES

## 8.1 Outcome measures

| Outcome | Definition |
| --- | --- |
| Prevalence of anemia | Proportion of Hb measurements < 11.0 g/dL. Anemia will be classified according to severity: mild (Hb 8.0 – 10.9), moderate (Hb 5.0 – 7.9), severe (Hb < 5.0). |
| Parasite prevalence | Proportion of study participants with a positive thick blood smear at the time of assessment |
| IRS coverage | Proportion of households sprayed |
| ITN coverage. | - Percentage of household with at least one bednet  - Percentage of household with at least one ITN  - Average number of nets per household  - Average number of ITNs per household  - Percentage of children age who slept under any net the prior night  - Percentage of children age who slept under an ITN the prior night  - Percentage of pregnant women who slept under any net the prior night  - Percentage of pregnant women who slept under an ITN the prior night |
| IPT coverage | Percentage of pregnant women on intermittent antimalarial treatment or antimalarial chemoprophylaxis |
| ACT’s coverage | Proportion of febrile episodes in children treated with an ACT |
| Prompt effective treatment of fever | Proportion of children with fever treated within 24 hours of onset of symptoms with an ACT |
| Under five Crude Death Rate (all cause mortality) | The number of children who die by the age of five, per thousand live births. |
| Under five malaria mortality rate | Proportion of deaths in children under five attributed to malaria per thousand live births. |
| Prevalence of malaria specific immune responses | Proportion of study participants with malaria specific immune responses at the time of assessment  Antibody density in participants at the time of assessment |

## 8.2 Analytical plan and sample size/power calculations

Statistical analysis will be undertaken using Stata 10 (Stata Corp., College Station, TX, USA). The prevalence of malaria infection will be based on the thin and thick blood smear readings. This procedure will enable estimation of parasite density, differentiation of Plasmodium species and identification of gametocytes. The proportion of children infected with single and multiple parasite species and the mean intensity of infection/parasite density will be calculated. Differences in proportions by age, sex and transmission setting will be assessed by logistic regression and differences in means using a Student t-test (for parametric data) and a Mann-Whitney test (for non-parametric data). Prevalence of infection and anemia, and coverage rates will be presented as frequencies with appropriate 95% binomial confidence intervals. The report and other related documents will be extensively disseminated to stakeholders. Avenues for dissemination will include publications, working papers, and atlases.

Infection. The primary metric used to measure infection for both the gold standard method (community surveys) and streamlined method (school surveys) will be the parasite rate (PR), defined as the number of positive blood smears divided by the number of smears examined. For the gold standard method, PR will be measured using the cross-sectional surveys of an estimated 300 children (200 households x 1.5 children per household) aged 6-14 years from the community surveys. For the streamlined method, PR will be estimated using cross-sectional surveys of 300 children (3 schools x 100 children per school) aged 6-14 years from the primary school surveys.

We will test the hypothesis that estimates of PR will be similar using the two methodologies. Point estimates of PR will be calculated for each biannual survey and compared using the McNemar’s test of paired proportions. Assuming that we will have a sample size of 300 children per survey for each methodology, we will have 80% power (one-sided significance level of 0.05) to reject the alternative hypothesis that there is a difference in estimates of PR assuming that the absolute difference in PR is no greater than 10% across a range of PRs in the community surveys of 20-80%. Whether or not these analyses show any evidence of significant differences between the methods, we will explore whether there is any systematic bias in the estimate of the streamlined method compared to the gold standard method (e.g. the streamlined method consistently over- or underestimates PR). Ecological logistic regression models will be used to predict the result of the gold standard method using data from the streamlined method, allowing adjustment for bias. Final models will then be prospectively validated in independent test sets to insure their accuracy.

Mortality. The primary metric for malaria mortality will be the relative change in absolute measures of malaria mortality. For the gold standard method, the measure of malaria morality will be the number of malaria-associated deaths in children under 5 years of age per 1,000 live births. Data on malaria mortality using the gold standard method will come from the biannual community surveys and verbal autopsy. For the streamlined method, the measure of malaria morality will be the number of malaria-associated deaths in children under 5 years of age captured by our inpatient surveillance system (described in another protocol).

We will test the hypothesis that relative changes in malaria mortality will be similar using the two methodologies. Point estimates of relative mortality from one year to the next will be calculated and compared, by calculating the variance of the streamlined method using the delta method and the variance of the gold standard method using a bootstrap test. Assuming that we will have a sample size of 800 children per year from the community surveys with baseline mortality rates of 6.5, 12.9, and 19.4 per 1000 (based on 2006 Uganda DHS survey and assuming malaria-attributable mortality of 20%, 40%, and 60%), and baseline number of malaria-associated deaths from our inpatient surveillance of 12, 60, and 84 (based on preliminary inpatient surveillance data) for low, medium, and high transmission sites respectively, we will have 80% power (one-sided significance level of 0.05) to reject the alternative hypothesis that there is an absolute difference in estimates of relative mortality of 1.30, 0.50 and 0.41 for low, medium, and high transmission sites, respectively. Whether or not these analyses show any evidence of significant differences between the methods, we will explore whether there is any systematic bias in the estimate of the streamlined method compared to the gold standard method (e.g. the streamlined method consistently over- or underestimates relative mortality). Log-linear regression models will be used to predict the result of the gold standard method using data from the streamlined method, allowing adjustment for bias.

Immunology. The age-dependent SCR will be determined for each study area at each time-point and related to the concurrent parasite prevalence by RDT, microscopy and PCR and anemia prevalence.

Evidence for a change in transmission intensity will be assessed by fitting more than one force of infection to age-dependent antibody prevalence data. Antibody prevalence and density in school children will be related to the concurrent parasite prevalence by RDT, microscopy and PCR and anemia prevalence.

Decision on the optimal methods for surveillance. If any of the streamlined methods described above do not approximate the corresponding gold standard methods and attempts at correcting systematic bias are not reliable (e.g. unable to consistently predict estimates using the gold standard method) then we will conclude that the streamlined method is not a reasonable substitute for the gold standard method.

# 9.0 DATA MANAGEMENT

All data will be collected by survey teams using hand-held computers (tablets). Prior to conducting the surveys information from the questionnaires and fields for entering results of biomarker testing will be programmed into the hand-held computers. Programming will include range checks, structure checks and internal consistency checks. Before leaving the household or schools, an inventory will be made of the completed questionnaires and blood samples collected; both will be checked to make sure they are labelled correctly. The completed questionnaires will be checked for mistakes and completeness. Where eligible respondents are absent from the home or school, a minimum of three additional call back visits will be made on different days. Data from these devices will be transferred at the end of every day to our data core facility in Kampala and stored on a secure server. The data file will be kept on a separate network so that only authorized survey staff will have access to the data during collection and processing phase. The file with data from the questionnaires will be merged with results from reading the malaria slides at the laboratory, using the unique bar codes. All filter paper samples and blood slides will be returned to UMSP offices in Kampala.

# 10.0 ETHICAL CONSIDERATIONS

## 10.1 Institutional Review Boards

This protocol and the informed consent documents will be reviewed and approved by all institutional review boards (IRBs) before the study begins. Any amendments or modifications to this material will also be reviewed and approved by the IRBs prior to implementations. The IRBs will include Makerere University School of Medicine Research and Ethics Committee (SOMREC), Uganda National Council of Science and Technology (UNCST), London School of Hygiene & Tropical Medicine (LSHTM) Ethics Committee and University of California, San Francisco, Committee on Human Research (UCSF CHR)

## 10.2 Informed consent procedures

Written informed consent will be sought from respondents for all aspects of the survey. The informed consent statements will contain all of the information the respondent needs to make an informed decision about whether or not to participate in the survey. The consent and assent forms to be used in the study will be translated into the locally used languages used at the study sites and also back-translated into English to check for any loss or change of meaning. Japadhola and Swahili will be used in Nagongera, Lusoga in Walukuba while Rukiga and Runyakole will be used in Kihihi. The informed consent and assent discussion will be conducted by the study interviewers in an appropriate language that the participant’s understand and a translator will be used if necessary. If a study participant or their parent or guardian (for children) is unable to read or write, their fingerprint will substitute for a signature, and a signature from a witness to the informed consent procedures will be obtained.

## 10.3 Confidentiality of respondents’ information

The protocol has been designed to protect the confidentiality of survey respondents. Interviewers will be instructed to conduct interviews with women in private with no other adults present, unless specifically requested by the respondent. Training will emphasize the importance of keeping all survey answers strictly confidential. A high level of confidentiality also will be strictly adhered to in handling the data from the survey. Names of individual respondents will not be entered into the computerized data file. A random error factor will also be introduced to the geographic positioning coordinates for each sample point so as to disable the ability to locate any particular point accurately. Unique identification numbers will be assigned to each household and woman respondent, as well as to those from whom biomarker data are collected, and will be used in the analysis. The Survey Directors who are fully knowledgeable about all aspects of human participant protection will be responsible for transferring such knowledge and concern to the field teams. In addition, field supervisors will ensure that confidentiality is maintained during the implementation of fieldwork.

## 10.4 Potential benefits to participants

Several biological tests will be conducted in the field and results returned to the respondents and where feasible treatment, care or follow-up will be provided. Test results for anemia (indicating whether the child has non-severe anemia or severe anemia and test results for malaria with Rapid Diagnostic Tests will be given in the field. Those with severe anemia or severe malaria will be referred for medical treatment. Respondents testing positive for malaria with Rapid Diagnostic Tests will be offered a free full-course treatment with the recommended antimalarials.

## 10.5 Potential risks to participants

The risk to participants from taking part in the interview portion of the survey is minimal. Finger pricks or heel pricks for blood collection from children may inflict slight pain. Since the survey will use sterile equipment, there will be minimal risk of infection. Laboratory technicians responsible for taking finger stick capillary blood samples will use disposable gloves, alcohol swabs, sterile gauze, and disposable lancets so as to eliminate risk of contamination. The area from which blood is drawn will be thoroughly cleaned with an alcohol prep swab before the puncture is made. After the blood has been collected, the blood flow will be stopped by applying pressure on the puncture site with a gauze pad. Once the bleeding stops, the area will be protected from infection by a band-aid. All persons conducting the blood testing will be specifically trained in these procedures. In respect of the malaria treatment, there is a reported small risk of adverse effects (typically around 3%) with artemether-lumefantrine, which include dizziness, fatigue, lack of appetite and palpitations. Parents of children provided with the treatment will be informed of these potential adverse effects and instructed on simple measures or to attend a nearest facility to counteract/treat them. Parents will be asked about their child’s medical treatment history and particularly whether they have allergy to any drugs.

## 10.6 Potential risks to study staff

The risk to field workers participating in the interview portion of the survey is minimal. Standard precautionary measures will be taken including hand washing, protective gloves and correct disposal of sharps. Disposable lancets will be used for finger/heel pricks which will minimize the possibility of accidental needle pricks, however, if it happens, those affected will be referred to the nearest regional referral hospital for PEP (post-exposure prophylaxis). All referral hospitals in Uganda offer PEP services. The field staff will be trained on proper collection of blood samples and on the emergency care procedures after an accidental needle prick.

## 10.7 Reimbursement and incentives

Respondents will not receive any compensation or payment.

# REFERENCES.

(1) Breman JG. Eradicating malaria. Sci Prog 2009;92(Pt 1):1-38.

(2) Okello PE, Van BW, Byaruhanga AM, Correwyn A, Roelants P, Talisuna A et al. Variation in malaria transmission intensity in seven sites throughout Uganda. Am J Trop Med Hyg 2006 August;75(2):219-25.

(3) WHO. World Malaria Report 2009. 2019.

(4) Lynch KI, Beach R, Asamoa K, Adeya G, Nambooze J and Janowsky E. President's Malaria Initiative, Rapid Assessment Report - Uganda. 2005.

(5) Uganda Malaria Indicator Survey, 2009. 2010.

(6) The Abuja Declaration and the Plan of Action, Abuja, 25^th^ April . 2000.

(7) Uganda Malaria Control Strategic Plan 2005/6-2009/10 . 2005.

(8) PMI Uganda. **Malaria Operational Plan for FY 2010** . 2010.

(9) UBOS and Macro International Inc. Uganda Bureau of Statistics (UBOS) and macro International Inc. 2007. Uganda Demographic and Health Survey 2006. Calverton, Maryland, USA. 2007.

(10) Brabin BJ. An analysis of malaria in pregnancy in Africa. Bull World Health Organ 1983;61(6):1005-16.

(11) Hviid L, Staalsoe T. Malaria immunity in infants: a special case of a general phenomenon? Trends Parasitol 2004 February;20(2):66-72.

(12) Clyde DF. Treatment of multi-drug resistant malaria. East Afr Med J 1967 June;44(6):231-7.

(13) DRAPER CC, SMITH A. Malaria in the Pare area of Tanganyika. Part II. Effects of three years' spraying of huts with dieldrin. Trans R Soc Trop Med Hyg 1960 July;54:342-57.:342-57.

(14) Onori E. Distribution of Plasmodium ovale in the eastern, western and northern regions of Uganda. Bull World Health Organ 1967;37(4):665-8.

(15) Hay SI, Smith DL, Snow RW. Measuring malaria endemicity from intense to interrupted transmission. Lancet Infect Dis 2008 June;8(6):369-78.

(16) Smith DL, Dushoff J, Snow RW, Hay SI. The entomological inoculation rate and Plasmodium falciparum infection in African children. Nature 2005 November 24;438(7067):492-5.

(17) Smith DL, Guerra CA, Snow RW, Hay SI. Standardizing estimates of the Plasmodium falciparum parasite rate. Malar J 2007 September 25;6:131.:131.

(18) Snow RW, Craig M, Deichmann U, Marsh K. Estimating mortality, morbidity and disability due to malaria among Africa's non-pregnant population. Bull World Health Organ 1999;77(8):624-40.

(19) Soleman N, Chandramohan D, Shibuya K. Verbal autopsy: current practices and challenges. Bull World Health Organ 2006 March;84(3):239-45.

(20) Breman JG. The ears of the hippopotamus: manifestations, determinants, and estimates of the malaria burden. Am J Trop Med Hyg 2001 January;64(1-2 Suppl):1-11.

(21) Setel PW, Sankoh O, Rao C, Velkoff VA, Mathers C, Gonghuan Y et al. Sample registration of vital events with verbal autopsy: a renewed commitment to measuring and monitoring vital statistics. Bull World Health Organ 2005 August;83(8):611-7.

(22) Quigley MA, Chandramohan D, Rodrigues LC. Diagnostic accuracy of physician review, expert algorithms and data-derived algorithms in adult verbal autopsies. Int J Epidemiol 1999 December;28(6):1081-7.

(23) Quigley MA. Commentary: verbal autopsies--from small-scale studies to mortality surveillance systems. Int J Epidemiol 2005 October;34(5):1087-8.

(24) World Health Organization. A standard verbal autopsy method for investigating causes of death in infants and children, 1999; WHO/CDS/CSR/ISR/99.4.

(25) Bhattarai A, Ali AS, Kachur SP, Martensson A, Abbas AK, Khatib R et al. Impact of artemisinin-based combination therapy and insecticide-treated nets on malaria burden in Zanzibar. PLoS Med 2007 November 6;4(11):e309.

(26) Ceesay SJ, Casals-Pascual C, Erskine J, Anya SE, Duah NO, Fulford AJ et al. Changes in malaria indices between 1999 and 2007 in The Gambia: a retrospective analysis. Lancet 2008 November 1;372(9649):1545-54.

(27) O'Meara WP, Bejon P, Mwangi TW, Okiro EA, Peshu N, Snow RW et al. Effect of a fall in malaria transmission on morbidity and mortality in Kilifi, Kenya. Lancet 2008 November 1;372(9649):1555-62.

(28) Hsiang MS, Lin M, Dokomajilar C, Kemere J, Pilcher CD, Dorsey G, Greenhouse B. PCR-based pooling of dried blood spots for detection of malaria parasites: optimization and application to a cohort of Ugandan children. J Clin Microbiol 2010 Oct;48(10):3539-43.

(29) Drakeley CJ, Corran PH, Coleman PG, Tongren JE, McDonald SL, Carneiro I, Malima R, Lusingu J, Manjurano A, Nkya WM, Lemnge MM, Cox J, Reyburn H, Riley EM. Estimating medium- and long-term trends in malaria transmission by using serological markers of malaria exposure. Proc Natl Acad Sci USA 2005 Apr 5;101914):5108-13.

# Appendices.

Appendix 1: Household and women’s survey - consent to participate in a research study

Appendix 2: Household and women’s survey – assent to participate in a research study

Appendix 3: Clinical survey – consent to participate in a research study (for children and adults)

Appendix 4: Clinical survey – consent for future use of biological specimens (for children and adults)

Appendix 5: Clinical survey – assent to participate in a research study (for children over 7 years)

Appendix 6: School survey – consent to participate in a research study

Appendix 7: School survey – consent for future use of biological specimens

Appendix 8: School survey – assent for children over 8 years of age

Appendix 9: Community Survey: Household Survey Questionnaire Data Dictionary

Appendix 10: Community Survey: Woman’s Questionnaire Data Dictionary

Appendix 11: Community Survey: Verbal Autopsy – Neonatal module - Data Dictionary

Appendix 12: Community Survey: Verbal Autopsy – Neonatal module - Data Dictionary

Appendix 13: School Survey: Data Dictionary
